# Oral toxicity of titanium dioxide P25 at repeated dose 28-day and 90-day in rats

**DOI:** 10.1186/s12989-020-00350-6

**Published:** 2020-07-17

**Authors:** Min Beom Heo, Minjeong Kwak, Kyu Sup An, Hye Jin Kim, Hyeon Yeol Ryu, So Min Lee, Kyung Seuk Song, In Young Kim, Ji-Hwan Kwon, Tae Geol Lee

**Affiliations:** 1 Center for Nano-Bio Measurement, Industrial Metrology, Korea Research Institute of Standards and Science (KRISS), Yuseong-Gu, Daejeon, Republic of Korea; 2 Korea Conformity Laboratories (KCL), Yeonsu-Gu, Incheon, Republic of Korea; 3 Center for Nanocharacterization, Industrial Metrology, Korea Research Institute of Standards and Science (KRISS), Yuseong-Gu, Daejeon, Republic of Korea

**Keywords:** TiO_2_ nanoparticles, Oral toxicity, NOAEL, Subchronic oral exposure

## Abstract

**Background:**

Nanotechnology is indispensable to many different applications. Although nanoparticles have been widely used in, for example, cosmetics, sunscreen, food packaging, and medications, they may pose human safety risks associated with nanotoxicity. Thus, toxicity testing of nanoparticles is essential to assess the relative health risks associated with consumer exposure.

**Methods:**

In this study, we identified the NOAEL (no observed adverse effect level) of the agglomerated/aggregated TiO_2_ P25 (approximately 180 nm) administered at repeated doses to Sprague-Dawley (SD) rats for 28 and 90 days. Ten of the 15 animals were necropsied for toxicity evaluation after the repeated-dose 90-day study, and the remaining five animals were allowed to recover for 28 days. The agglomerated/aggregated TiO_2_ P25 dose levels used included 250 mg kg^− 1^ d^− 1^ (low), 500 mg kg^− 1^ d^− 1^ (medium), and 1000 mg kg^− 1^ d^− 1^ (high), and their effects were compared with those of the vehicle control. During the treatment period, the animals were observed for mortality, clinical signs (detailed daily and weekly clinical observations), functional observation battery, weekly body weight, and food and water consumption and were also subjected to ophthalmological examination and urinalysis. After termination of the repeated-dose 28-day, 90-day, and recovery studies, clinical pathology (hematology, blood coagulation time, and serum biochemistry), necropsy (organ weights and gross findings), and histopathological examinations were performed.

**Results:**

No systemic toxicological effects were associated with the agglomerated/aggregated TiO_2_ P25 during the repeated-dose 28-day, 90-day, and recovery studies in SD rats. Therefore, the NOAEL of the agglomerated/aggregated TiO_2_ P25 was identified as 1000 mg kg^− 1^ d^− 1^, and the substance was not detected in the target organs.

**Conclusion:**

Subacute and subchronic oral administration of the agglomerated/aggregated TiO_2_ P25 was unlikely to cause side effects or toxic reactions in rats.

## Background

Human exposure to nanoparticles is increasing as these materials are used in numerous applications and commercial products. Given their small size, nanoparticles can penetrate tissues and cells, inducing toxic effects. Currently, TiO_2_ is one of the most frequently used nanomaterials. In particular, micro- or nanoscale TiO_2_ has been widely used commercially in cosmetics and skin care products, paints, plastics, paper, toothpicks, and other products [1, 2]. Nanoscale TiO_2_ represents less than 2% of total consumption and presents physical properties different from microscale TiO_2_. For example, nanoscale TiO_2_ is an efficient photocatalyst used in products such as dye-sensitized solar cells and UV protection agents. Pigment-grade TiO_2_, which accounts for more than 98% of total consumption, has a bright white color, and is often used to enhance the appearance of food [3]. E171 (food-grade TiO_2_), used as food additive, does not have a surface coating and may include some nanoparticles resulting from the grinding process, a conventional physical treatment. The United States Food and Drug Administration (FDA) approved pigment-grade TiO_2_ as a human food coloring agent in 1966 with the stipulation that the TiO_2_ levels are lower than 1% of the food weight [4]. According to a recent EFSA opinion article, six different brands of E171 containing different percentages of nanoparticles are used in food in the EU. The panel proposed changes to the current specifications, referring that E171 should have a minimal external dimension of 100 nm, equivalent to less than 50% of the number of constituent particles with a median minimal external dimension below 100 nm [5].

A number of recent studies have characterized the mammalian toxicity of TiO_2_. In particular, absorbed TiO_2_ nanoparticles were found to be toxic to various organs because they induce oxidative stress [6, 7]. Furthermore, other research studies have also found that TiO_2_ nanoparticles are distributed to the major organs after inhalation [8], oral ingestion [9], and dermal penetration [10] and may translocate to systemic organs from the lung and gastrointestinal tract (GIT) [11]. Another example is that rats exposed to high levels of fine TiO_2_ particles by inhalation for two years developed lung tumors [12]. Based on such findings, TiO_2_ nanoparticles were categorized by the International Agency for Research on Cancer (IARC) as a Group 2B carcinogen (possibly carcinogenic to humans) [13]. Nevertheless, there have also been studies reporting low toxicity of TiO_2_. Specifically, some reports have shown that oral ingestion of TiO_2_ allows it to pass through the body unabsorbed because of particle agglomeration in the GIT [14, 15].

TiO_2_ P25, which is covered in this paper, is crystallized at an 80:20 ratio of anatase and rutile and has excellent photocatalytic function without surface coating, so it is generally used as a catalyst. Many toxicity studies on TiO_2_ P25 have focused on inhalation and skin exposure, but studies on oral exposure have been not been reported. Therefore, the aim of the present study was to evaluate the toxicity of the agglomerated/aggregated TiO_2_ P25 in rats following subchronic oral exposure. The results of two independent studies are condensed in this report and include repeated-dose 28-day and 90-day oral toxicity studies and a 28-day recovery study in rats.

## Results

### Characterization of TiO_2_ nanoparticles

**Fig. 1:**
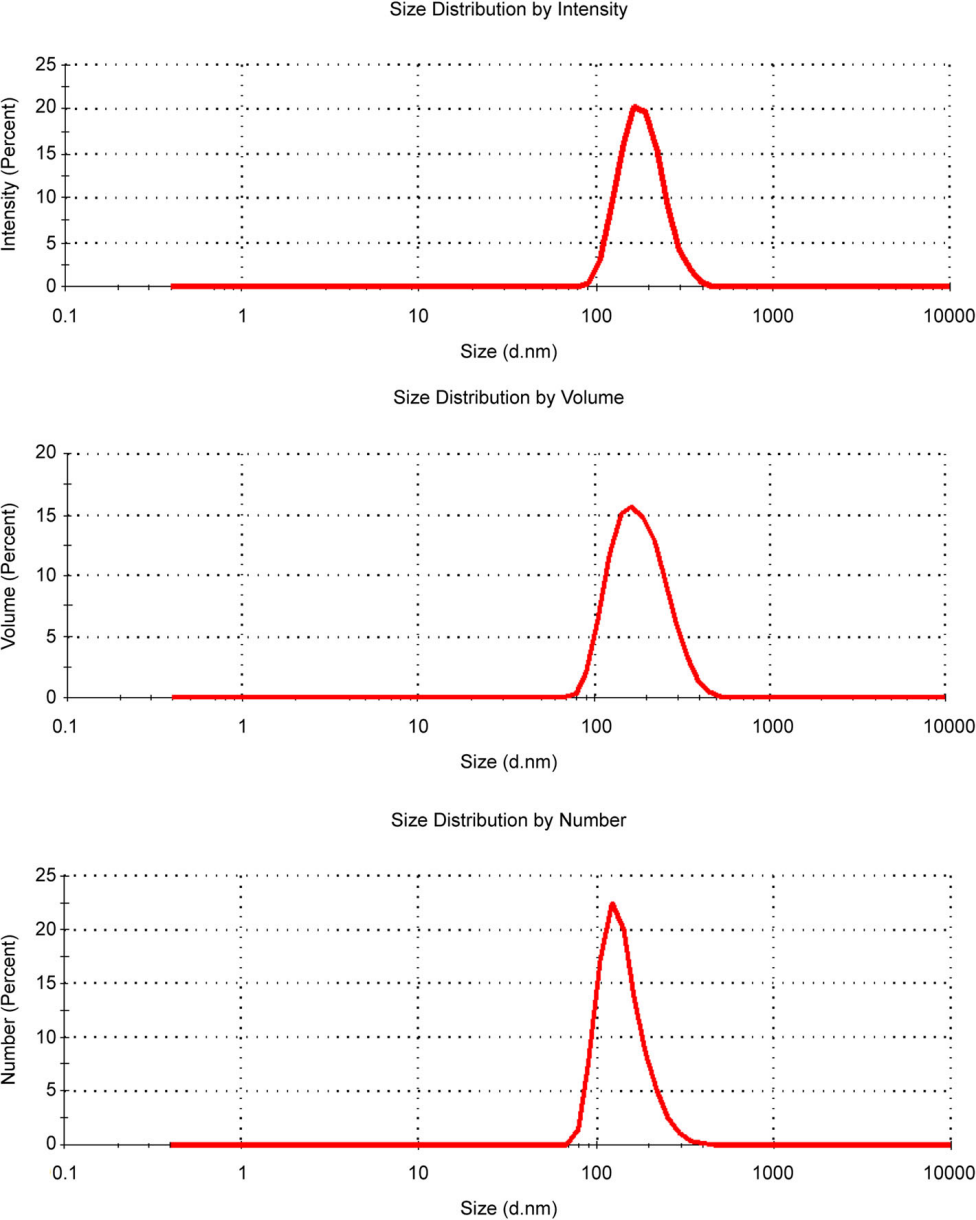
Hydrodynamic diameter distribution of the agglomerated/aggregated TiO_2_ P25 using dynamic light scattering (DLS) characterization

**Fig. 2:**
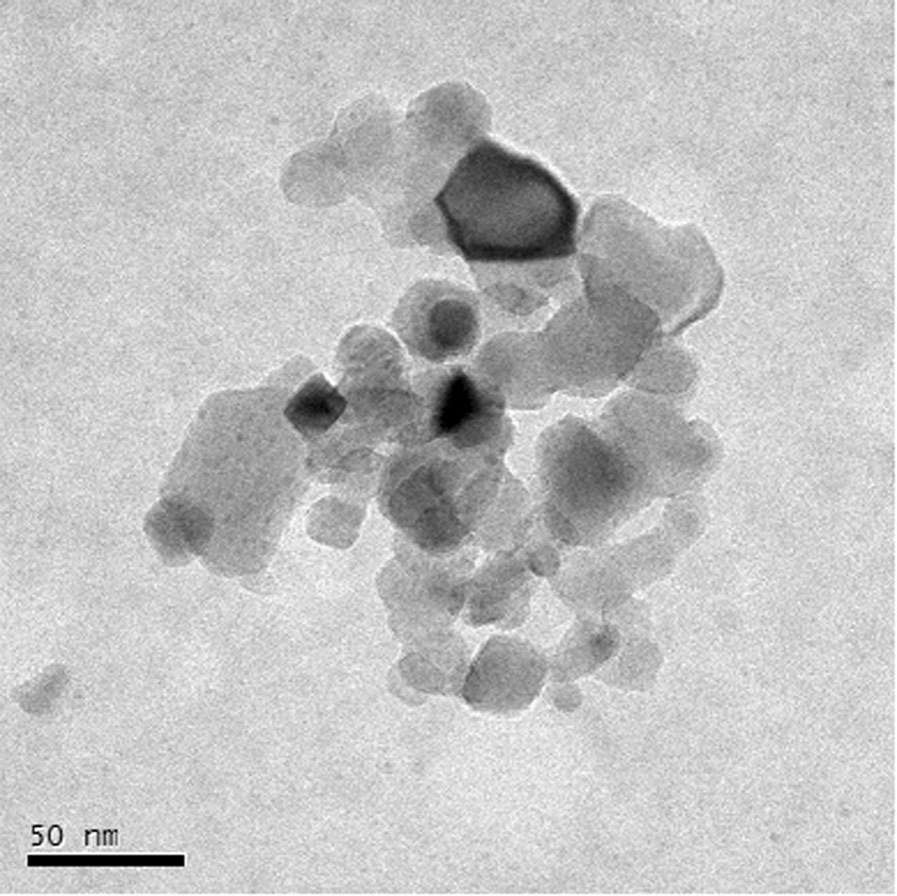
Transmission electron microscopy (TEM) image of the agglomerated/aggregated TiO_2_ P25

The particle size distribution of the nanoparticle suspension was determined by dynamic light scattering (DLS). After dispersing the nanoparticles in 5 mM sodium phosphate buffer, the size ranges of the agglomerated/aggregated TiO_2_ P25 were 187.3 ± 54.04 nm (intensity), 188.7 ± 67.97 nm (volume), and 142.9 ± 43.97 nm (number), with a polydispersity index of 0.22 (Fig. 1). The particle sizes were also confirmed using transmission electron microscopy (TEM) and are in agreement with those obtained by DLS (Fig. 2).

### Determination of the repeated 28-day oral dose range of TiO_2_

**Fig. 3:**
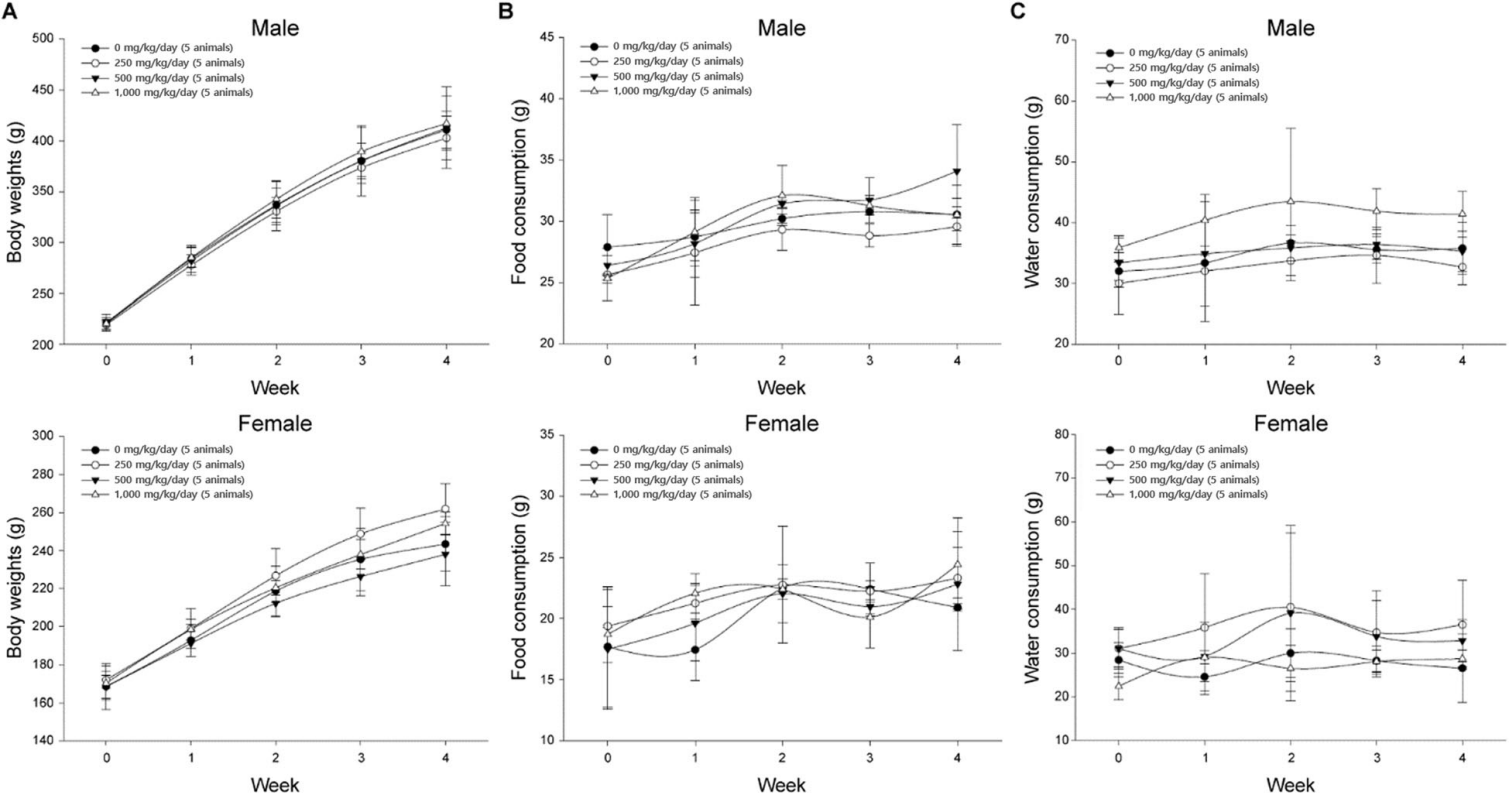
Body weights and daily food and water consumption on the repeated-dose 28-day oral toxicity study. **(a)** Body weights of male and female rats, **(b)** Mean daily food consumption of male and female rats, **(c)** Mean daily water consumption of male and female rats

We evaluated the no observed adverse effect level (NOAEL) for the repeated-dose oral toxicity of agglomerated/aggregated TiO_2_ P25 by administering the substance to SD rats for 28 days and determining the dose levels in a repeated-dose oral toxicity 90-day study. No mortality or abnormal clinical signs were detected during the experimental period (data not shown). There were no statistically significant differences between the treatment and vehicle control groups in terms of body weight (Fig. 3a) or food and water consumption (Fig. 3b and c) of the male and female rats during the experimental period. The hematology results (Table 1) reveled the following changes relative to the vehicle control group: lower LY counts for the female 250 mg kg^− 1^, 500 mg kg^− 1^, and 1000 mg kg^− 1^ dosing groups (*P* < 0.05) and higher MCV in the female 250 mg kg^− 1^ dosing group (*P* < 0.05). Nevertheless, these differences did not indicate a significant dose-response relationship. Based on hematologic historical data accumulated in our laboratory on 10-week-old female rats, the range of WBC and LY ranged from 2.79 to 9.33 K/μL and 2.17 to 8.15 K/μL (95% confidence interval range). WBC and LY values of all test substance groups fall within the normal range. As exceptionally high values in the control group were measured continuously, the values of the test substance group decrease relatively. As for the serum biochemistry results (Table 2), comparatively high Cl levels were observed in the female 500 mg kg^− 1^ dosing group (*P* < 0.01), but this difference was determined to be unrelated to the treatment because no dose-response relationship was detected. No ocular abnormalities were detected in any of the animals (data not shown). Urinalysis indicated significant differences among the male rats in terms of SG level (*P* < 0.05) (Table S1). However, these discrepancies were not attributed to the treatment because no distinct dose-response relationship was found.

**Table 1:** Hematological values of rats in the repeated-dose 28-day study, mean ± SD (number of animals)

Summary of hematological tests from the repeated-dose 28-day study																
Sex: Male																
Test item	Group(mg/kg/day)															
	G1(0)				G2(250)				G3(500)				G4(1000)			
WBC^1^(K/μL)	**7**	**±**	**2.06**	**(5)**	**8.09**	**±**	**2.03**	**(5)**	**6.39**	**±**	**1.94**	**(5)**	**7.15**	**±**	**1.29**	**(5)**
PMN^2^(K/μL)	**1.1**	**±**	**0.44**	**(5)**	**1.17**	**±**	**0.25**	**(5)**	**0.81**	**±**	**0.35**	**(5)**	**1.29**	**±**	**0.58**	**(5)**
EO^3^(K/μL)	**0.07**	**±**	**0.03**	**(5)**	**0.06**	**±**	**0.02**	**(5)**	**0.06**	**±**	**0.04**	**(5)**	**0.06**	**±**	**0.03**	**(5)**
BA^4^(K/μL)	**0.01**	**±**	**0**	**(5)**	**0.01**	**±**	**0.01**	**(5)**	**0**	**±**	**0**	**(5)**	**0**	**±**	**0**	**(5)**
LY^5^(K/μL)	**5.66**	**±**	**1.63**	**(5)**	**6.69**	**±**	**1.91**	**(5)**	**5.34**	**±**	**1.54**	**(5)**	**5.59**	**±**	**1.11**	**(5)**
MO^6^(K/μL)	**0.14**	**±**	**0.06**	**(5)**	**0.11**	**±**	**0.01**	**(5)**	**0.12**	**±**	**0.1**	**(5)**	**0.14**	**±**	**0.04**	**(5)**
LUC^7^(K/μL)	**0.04**	**±**	**0.02**	**(5)**	**0.05**	**±**	**0.03**	**(5)**	**0.06**	**±**	**0.05**	**(5)**	**0.05**	**±**	**0.02**	**(5)**
PMNP^8^(%)	**15.5**	**±**	**3.9**	**(5)**	**15.1**	**±**	**4**	**(5)**	**12.5**	**±**	**2.6**	**(5)**	**18**	**±**	**7.1**	**(5)**
EOP^9^(%)	**0.9**	**±**	**0.2**	**(5)**	**0.7**	**±**	**0.2**	**(5)**	**0.8**	**±**	**0.4**	**(5)**	**0.8**	**±**	**0.3**	**(5)**
BAP^10^(%)	**0.1**	**±**	**0**	**(5)**	**0.1**	**±**	**0.1**	**(5)**	**0**	**±**	**0.1**	**(5)**	**0**	**±**	**0**	**(5)**
LYP^11^(%)	**81.1**	**±**	**4.4**	**(5)**	**82.1**	**±**	**4.2**	**(5)**	**83.9**	**±**	**4**	**(5)**	**78.4**	**±**	**7.3**	**(5)**
MOP^12^(%)	**1.9**	**±**	**0.5**	**(5)**	**1.4**	**±**	**0.4**	**(5)**	**1.8**	**±**	**1.1**	**(5)**	**2**	**±**	**0.3**	**(5)**
LUP^13^(%)	**0.5**	**±**	**0.2**	**(5)**	**0.7**	**±**	**0.3**	**(5)**	**0.8**	**±**	**0.5**	**(5)**	**0.7**	**±**	**0.2**	**(5)**
RBC^14^(M/μL)	**7.89**	**±**	**0.45**	**(5)**	**7.82**	**±**	**0.83**	**(5)**	**7.65**	**±**	**0.49**	**(5)**	**7.55**	**±**	**0.55**	**(5)**
Hb^15^(g/dL)	**15.5**	**±**	**0.8**	**(5)**	**13.3**	**±**	**2.5**	**(5)**	**14.7**	**±**	**0.8**	**(5)**	**14.6**	**±**	**0.6**	**(5)**
RDW^16^(%)	**11.2**	**±**	**0.4**	**(5)**	**11.3**	**±**	**0.3**	**(5)**	**11.2**	**±**	**0.4**	**(5)**	**11.8**	**±**	**1**	**(5)**
HCT^17^(%)	**46.8**	**±**	**2.9**	**(5)**	**46.1**	**±**	**3.6**	**(5)**	**45.1**	**±**	**2.3**	**(5)**	**44.4**	**±**	**2.7**	**(5)**
MCV^18^(fL)	**59.3**	**±**	**1.8**	**(5)**	**59.1**	**±**	**3.6**	**(5)**	**59.1**	**±**	**1.6**	**(5)**	**58.8**	**±**	**2.3**	**(5)**
MCH^19^(pg)	**19.6**	**±**	**0.5**	**(5)**	**17.4**	**±**	**4.3**	**(5)**	**19.2**	**±**	**0.6**	**(5)**	**19.3**	**±**	**1**	**(5)**
MCHC^20^(g/dL)	**33.1**	**±**	**0.7**	**(5)**	**29.3**	**±**	**6.7**	**(5)**	**32.5**	**±**	**0.3**	**(5)**	**32.8**	**±**	**0.8**	**(5)**
Reti^21^(%)	**2.6**	**±**	**0.3**	**(5)**	**2.97**	**±**	**0.57**	**(5)**	**2.65**	**±**	**0.27**	**(5)**	**3.99**	**±**	**2.72**	**(5)**
PLT^22^(K/μL)	**1068**	**±**	**110**	**(5)**	**1081**	**±**	**136**	**(5)**	**1127**	**±**	**193**	**(5)**	**1162**	**±**	**111**	**(5)**
MPV^23^(fL)	**6.9**	**±**	**0.3**	**(5)**	**6.8**	**±**	**0.8**	**(5)**	**7**	**±**	**0.3**	**(5)**	**7.2**	**±**	**0.1**	**(5)**
Sex: Female																
Test item	Group(mg/kg/day)															
	G1(0)				G2(250)				G3(500)				G4(1000)			
WBC^1^(K/μL)	**11.87**	**±**	**3.21**	**(5)**	**7.09**	**±**	**0.6**	**(5)**	**6.99**	**±**	**1.99**	**(5)**	**8.08**	**±**	**2**	**(5)**
PMN^2^(K/μL)	**1.05**	**±**	**0.17**	**(5)**	**0.72**	**±**	**0.35**	**(5)**	**0.62**	**±**	**0.15**	**(5)**	**1**	**±**	**0.49**	**(5)**
EO^3^(K/μL)	**0.1**	**±**	**0.03**	**(5)**	**0.06**	**±**	**0**	**(5)**	**0.07**	**±**	**0.03**	**(5)**	**0.09**	**±**	**0.03**	**(5)**
BA^4^(K/μL)	**0.01**	**±**	**0.01**	**(5)**	**0**	**±**	**0.01**	**(5)**	**0**	**±**	**0**	**(5)**	**0**	**±**	**0.01**	**(5)**
LY^5^(K/μL)	**10.4**	**±**	**3.25**	**(5)**	6.08*	**±**	**0.74**	**(5)**	6.05*	**±**	**1.83**	**(5)**	6.68*	**±**	**1.87**	**(5)**
MO^6^(K/μL)	**0.19**	**±**	**0.04**	**(5)**	**0.16**	**±**	**0.06**	**(5)**	**0.16**	**±**	**0.04**	**(5)**	**0.19**	**±**	**0.1**	**(5)**
LUC^7^(K/μL)	**0.14**	**±**	**0.04**	**(5)**	**0.06**	**±**	**0.02**	**(5)**	**0.08**	**±**	**0.03**	**(5)**	**0.11**	**±**	**0.06**	**(5)**
PMNP^8^(%)	**9.5**	**±**	**3.3**	**(5)**	**10.3**	**±**	**5**	**(5)**	**9.1**	**±**	**1.6**	**(5)**	**12.7**	**±**	**5.5**	**(5)**
EOP^9^(%)	**0.9**	**±**	**0.3**	**(5)**	**0.8**	**±**	**0.1**	**(5)**	**1**	**±**	**0.4**	**(5)**	**1.1**	**±**	**0.2**	**(5)**
BAP^10^(%)	**0**	**±**	**0.1**	**(5)**	**0.1**	**±**	**0**	**(5)**	**0**	**±**	**0**	**(5)**	**0.1**	**±**	**0.1**	**(5)**
LYP^11^(%)	**86.8**	**±**	**3.9**	**(5)**	**85.6**	**±**	**5.8**	**(5)**	**86.4**	**±**	**2.1**	**(5)**	**82.4**	**±**	**6.7**	**(5)**
MOP^12^(%)	**1.6**	**±**	**0.5**	**(5)**	**2.4**	**±**	**0.9**	**(5)**	**2.3**	**±**	**0.3**	**(5)**	**2.4**	**±**	**1**	**(5)**
LUP^13^(%)	**1.1**	**±**	**0.1**	**(5)**	**0.9**	**±**	**0.2**	**(5)**	**1.1**	**±**	**0.2**	**(5)**	**1.4**	**±**	**0.4**	**(5)**
RBC^14^(M/μL)	**7.66**	**±**	**0.26**	**(5)**	**7.56**	**±**	**0.23**	**(5)**	**7.72**	**±**	**0.34**	**(5)**	**7.92**	**±**	**0.37**	**(5)**
Hb^15^(g/dL)	**14.4**	**±**	**0.6**	**(5)**	**14.7**	**±**	**0.4**	**(5)**	**14.7**	**±**	**0.4**	**(5)**	**15.1**	**±**	**0.6**	**(5)**
RDW^16^(%)	**10.4**	**±**	**0.2**	**(5)**	**10.4**	**±**	**0.5**	**(5)**	**10.4**	**±**	**0.4**	**(5)**	**10.4**	**±**	**0.1**	**(5)**
HCT^17^(%)	**42.2**	**±**	**1.6**	**(5)**	**43.5**	**±**	**1.1**	**(5)**	**43.1**	**±**	**0.9**	**(5)**	**44.9**	**±**	**2**	**(5)**
MCV^18^(fL)	**55**	**±**	**1.4**	**(5)**	57.5*	**±**	**0.5**	**(5)**	**55.9**	**±**	**1.6**	**(5)**	**56.7**	**±**	**1.1**	**(5)**
MCH^19^(pg)	**18.7**	**±**	**0.5**	**(5)**	**19.4**	**±**	**0.3**	**(5)**	**19.1**	**±**	**0.6**	**(5)**	**19.1**	**±**	**0.5**	**(5)**
MCHC^20^(g/dL)	**34**	**±**	**0.2**	**(5)**	**33.8**	**±**	**0.4**	**(5)**	**34.1**	**±**	**0.4**	**(5)**	**33.7**	**±**	**0.5**	**(5)**
Reti^21^(%)	**2.07**	**±**	**0.39**	**(5)**	**2.28**	**±**	**0.41**	**(5)**	**2.13**	**±**	**0.41**	**(5)**	**2.16**	**±**	**0.37**	**(5)**
PLT^22^(K/μL)	**1250**	**±**	**99**	**(5)**	**1070**	**±**	**107**	**(5)**	**1093**	**±**	**125**	**(5)**	**1206**	**±**	**179**	**(5)**
MPV^23^(fL)	**6.4**	**±**	**0.2**	**(5)**	**6.8**	**±**	**0.3**	**(5)**	**6.7**	**±**	**0.3**	**(5)**	**6.5**	**±**	**0.2**	**(5)**

1: White blood cell, 2: Neutrophil, 3: Eosinophil, 4: Basophil, 5: Lymphocyte, 6: Monocyte, 7: Large unstained cell, 8: Percent of neutrophil, 9: Percent of eosinophil, 10: Percent of basophil, 11: Percent of lymphocyte, 12: Percent of monocyte, 13: Percent of large unstained cell, 14: Red blood cell, 15: Hemoglobin, 16: Red cell distribution width, 17: Hematocrit, 18: Mean corpuscular volume, 19: Mean corpuscular hemoglobin, 20: Mean corpuscular hemoglobin concentration, 21: Reticulocyte, 22: Platelet, 23: Mean platelet volume

*: Significant difference compared with the control group, *P* < 0.05

**Table 2:** Biochemical analysis of rat serum in repeated-dose 28-day study, mean ± SD (number of animals)

Summary of serum biochemical tests from the repeated-dose 28-day study																
Sex: Male																
Test item	Group(mg/kg/day)															
	G1(0)				G2(250)				G3(500)				G4(1000)			
AST^1^(IU/L)	**110**	**±**	**25**	**(5)**	**112**	**±**	**22**	**(5)**	**111**	**±**	**30**	**(5)**	**123**	**±**	**27**	**(5)**
ALT^2^(IU/L)	**31**	**±**	**11**	**(5)**	**30**	**±**	**5**	**(5)**	**32**	**±**	**6**	**(5)**	**32**	**±**	**7**	**(5)**
GGT^3^(IU/L)	**0.8**	**±**	**0.45**	**(5)**	**1**	**±**	**0**	**(5)**	**1**	**±**	**0**	**(5)**	**1**	**±**	**0**	**(5)**
ALP^4^(IU/L)	**464**	**±**	**59**	**(5)**	**521**	**±**	**122**	**(5)**	**509**	**±**	**12**	**(5)**	**494**	**±**	**32**	**(5)**
BIL^5^(mg/dL)	**0.02**	**±**	**0.01**	**(5)**	**0.02**	**±**	**0.01**	**(5)**	**0.02**	**±**	**0.02**	**(5)**	**0.05**	**±**	**0.05**	**(5)**
BUN^6^(mg/dL)	**16.3**	**±**	**1.8**	**(5)**	**14.6**	**±**	**1.8**	**(5)**	**14.2**	**±**	**1.8**	**(5)**	**13.3**	**±**	**1.8**	**(5)**
CRE^7^(mg/dL)	**0.45**	**±**	**0.02**	**(5)**	**0.47**	**±**	**0.03**	**(5)**	**0.47**	**±**	**0.06**	**(5)**	**0.45**	**±**	**0.01**	**(5)**
UA^8^(mg/dL)	**0.9**	**±**	**0.2**	**(5)**	**0.9**	**±**	**0.1**	**(5)**	**1.1**	**±**	**0.3**	**(5)**	**1.1**	**±**	**0.1**	**(5)**
GLU^9^(mg/dL)	**148**	**±**	**30**	**(5)**	**137**	**±**	**15**	**(5)**	**124**	**±**	**26**	**(5)**	**149**	**±**	**30**	**(5)**
CHO^10^(mg/dL)	**77**	**±**	**21**	**(5)**	**60**	**±**	**14**	**(5)**	**73**	**±**	**17**	**(5)**	**67**	**±**	**19**	**(5)**
TG^11^(mg/dL)	**66**	**±**	**34**	**(5)**	**52**	**±**	**25**	**(5)**	**34**	**±**	**10**	**(5)**	**74**	**±**	**48**	**(5)**
PRO^12^(g/dL)	**5.9**	**±**	**0.2**	**(5)**	**5.8**	**±**	**0.2**	**(5)**	**5.8**	**±**	**0.3**	**(5)**	**5.9**	**±**	**0.2**	**(5)**
ALB^13^(g/dL)	**2.5**	**±**	**0.1**	**(5)**	**2.3**	**±**	**0.1**	**(5)**	**2.4**	**±**	**0.2**	**(5)**	**2.4**	**±**	**0.1**	**(5)**
A/G ratio^14^	**0.73**	**±**	**0.05**	**(5)**	**0.68**	**±**	**0.04**	**(5)**	**0.69**	**±**	**0.03**	**(5)**	**0.67**	**±**	**0.05**	**(5)**
LDH^15^(IU/L)	**1267**	**±**	**464**	**(5)**	**1129**	**±**	**494**	**(5)**	**1153**	**±**	**712**	**(5)**	**1320**	**±**	**656**	**(5)**
CPK^16^(U/L)	**692**	**±**	**219**	**(5)**	**674**	**±**	**243**	**(5)**	**714**	**±**	**382**	**(5)**	**696**	**±**	**321**	**(5)**
Ca^17^(mg/dL)	**9.7**	**±**	**0.4**	**(5)**	**9.8**	**±**	**0.5**	**(5)**	**9.7**	**±**	**0.5**	**(5)**	**9.7**	**±**	**0.4**	**(5)**
IP^18^(mg/dL)	**8.1**	**±**	**0.4**	**(5)**	**8.1**	**±**	**0.6**	**(5)**	**8.1**	**±**	**0.5**	**(5)**	**8.1**	**±**	**0.4**	**(5)**
Mg^19^(mg/dL)	**2.3**	**±**	**0.1**	**(5)**	**2.2**	**±**	**0.2**	**(5)**	**2.3**	**±**	**0.1**	**(5)**	**2.3**	**±**	**0.1**	**(5)**
Na^20^(mmol/L)	**148**	**±**	**2**	**(5)**	**149**	**±**	**2**	**(5)**	**148**	**±**	**1**	**(5)**	**147**	**±**	**1**	**(5)**
K^21^(mmol/L)	**5**	**±**	**0.4**	**(5)**	**5.1**	**±**	**0.2**	**(5)**	**4.9**	**±**	**0.3**	**(5)**	**4.8**	**±**	**0.3**	**(5)**
Cl^22^(mmol/L)	**98**	**±**	**2**	**(5)**	**100**	**±**	**2**	**(5)**	**99**	**±**	**2**	**(5)**	**99**	**±**	**2**	**(5)**
Sex: Female																
Test item	Group(mg/kg/day)															
	G1(0)				G2(250)				G3(500)				G4(1000)			
AST^1^(IU/L)	**113**	**±**	**13**	**(5)**	**94**	**±**	**15**	**(5)**	**116**	**±**	**15**	**(5)**	**102**	**±**	**10**	**(5)**
ALT^2^(IU/L)	**24**	**±**	**3**	**(5)**	**25**	**±**	**3**	**(5)**	**25**	**±**	**7**	**(5)**	**22**	**±**	**4**	**(5)**
GGT^3^(IU/L)	**1**	**±**	**0**	**(5)**	**0.8**	**±**	**0.45**	**(5)**	**1**	**±**	**0**	**(5)**	**1**	**±**	**0**	**(5)**
ALP^4^(IU/L)	**275**	**±**	**57**	**(5)**	**270**	**±**	**73**	**(5)**	**315**	**±**	**45**	**(5)**	**272**	**±**	**50**	**(5)**
BIL^5^(mg/dL)	**0.08**	**±**	**0.01**	**(5)**	**0.07**	**±**	**0.03**	**(5)**	**0.07**	**±**	**0.01**	**(5)**	**0.08**	**±**	**0.02**	**(5)**
BUN^6^(mg/dL)	**17.2**	**±**	**2.3**	**(5)**	**14.7**	**±**	**2.6**	**(5)**	**16**	**±**	**4.8**	**(5)**	**15**	**±**	**1.7**	**(5)**
CRE^7^(mg/dL)	**0.55**	**±**	**0.06**	**(5)**	**0.47**	**±**	**0.03**	**(5)**	**0.53**	**±**	**0.05**	**(5)**	**0.5**	**±**	**0.04**	**(5)**
UA^8^(mg/dL)	**1.2**	**±**	**0.2**	**(5)**	**0.9**	**±**	**0.2**	**(5)**	**0.9**	**±**	**0.1**	**(5)**	**1.1**	**±**	**0.2**	**(5)**
GLU^9^(mg/dL)	**117**	**±**	**15**	**(5)**	**133**	**±**	**31**	**(5)**	**118**	**±**	**11**	**(5)**	**128**	**±**	**14**	**(5)**
CHO^10^(mg/dL)	**67**	**±**	**6**	**(5)**	**83**	**±**	**12**	**(5)**	**70**	**±**	**11**	**(5)**	**76**	**±**	**22**	**(5)**
TG^11^(mg/dL)	**19**	**±**	**10**	**(5)**	**39**	**±**	**17**	**(5)**	**24**	**±**	**12**	**(5)**	**28**	**±**	**7**	**(5)**
PRO^12^(g/dL)	**6.5**	**±**	**0.3**	**(5)**	**6.7**	**±**	**0.3**	**(5)**	**6.1**	**±**	**0.3**	**(5)**	**6.5**	**±**	**0.3**	**(5)**
ALB^13^(g/dL)	**2.8**	**±**	**0.1**	**(5)**	**3**	**±**	**0.2**	**(5)**	**2.7**	**±**	**0.2**	**(5)**	**2.9**	**±**	**0.2**	**(5)**
A/G ratio^14^	**0.76**	**±**	**0.02**	**(5)**	**0.8**	**±**	**0.04**	**(5)**	**0.77**	**±**	**0.05**	**(5)**	**0.8**	**±**	**0.05**	**(5)**
LDH^15^(IU/L)	**1110**	**±**	**390**	**(5)**	**770**	**±**	**271**	**(5)**	**1094**	**±**	**416**	**(5)**	**986**	**±**	**371**	**(5)**
CPK^16^(U/L)	**665**	**±**	**203**	**(5)**	**476**	**±**	**176**	**(5)**	**605**	**±**	**157**	**(5)**	**573**	**±**	**177**	**(5)**
Ca^17^(mg/dL)	**10.1**	**±**	**0.3**	**(5)**	**10.2**	**±**	**0.3**	**(5)**	**9.9**	**±**	**0.4**	**(5)**	**10.3**	**±**	**0.3**	**(5)**
IP^18^(mg/dL)	**7.5**	**±**	**0.5**	**(5)**	**7.2**	**±**	**0.5**	**(5)**	**7.4**	**±**	**0.7**	**(5)**	**7.5**	**±**	**1**	**(5)**
Mg^19^(mg/dL)	**2.4**	**±**	**0.1**	**(5)**	**2.3**	**±**	**0.1**	**(5)**	**2.4**	**±**	**0**	**(5)**	**2.4**	**±**	**0.1**	**(5)**
Na^20^(mmol/L)	**146**	**±**	**1**	**(5)**	**145**	**±**	**2**	**(5)**	**144**	**±**	**2**	**(5)**	**146**	**±**	**1**	**(5)**
K^21^(mmol/L)	**4.7**	**±**	**0.2**	**(5)**	**4.6**	**±**	**0.2**	**(5)**	**4.5**	**±**	**0.1**	**(5)**	**4.6**	**±**	**0.4**	**(5)**
Cl^22^(mmol/L)	**98**	**±**	**2**	**(5)**	**100**	**±**	**1**	**(5)**	102**	**±**	**2**	**(5)**	**99**	**±**	**1**	**(5)**

1: Aspartate aminotransferase, 2: Alanine aminotransferase, 3: Gamma(γ)-glutamyl transferase, 4: Alkaline phosphatase, 5: Total bilirubin, 6: Blood urea nitrogen, 7: Creatinine, 8: Uric acid, 9: Glucose, 10: Total cholesterol, 11: Triglyceride, 12: Total protein, 13: Albumin, 14: Albumin/Globulin ratio, 15: Lactate dehydrogenase, 16: Creatine phosphokinase, 17: Calcium, 18: Inorganic phosphorus, 19: Magnesium, 20: Sodium, 21: Potassium, 22: Chloride

**: Significant difference compared with the vehicle control group, *P* < 0.01

There were no abnormal gross findings in any of the animals at necropsy (Table S2). In terms of organ weights (Table S3), the right adrenal glands were relatively lighter in the male dosing groups (*P* < 0.05) (absolute weights for 250 mg kg^− 1^ and 1000 mg kg^− 1^ and relative weights for 250 mg kg^− 1^, 500 mg kg^− 1^, and 1000 mg kg^− 1^). On the other hand, no substantial dose-response relationship or correlation between weight changes in bilateral organs were identified. Moreover, all measurements fell within the normal ranges. The increase in absolute liver weight of the female 250 mg kg^− 1^ group (*P* < 0.05) did not present an evident dose-response relationship, and these changes were not attributable to the test substance.

In summary, there were no observed systemic toxicological effects associated with the agglomerated/aggregated TiO_2_ P25 in the repeated-dose 28-day oral toxicity study on SD rats under the experimental conditions used. Therefore, a NOAEL for the agglomerated/aggregated TiO_2_ P25 of 1000 mg kg^− 1^ was identified, and a maximum dose of 1000 mg kg^− 1^ d^− 1^ was considered acceptable for the repeated-dose 90-day oral toxicity study.

### Repeated-dose 90-day oral toxicity and 28-day recovery studies of TiO_2_

**Fig. 4:**
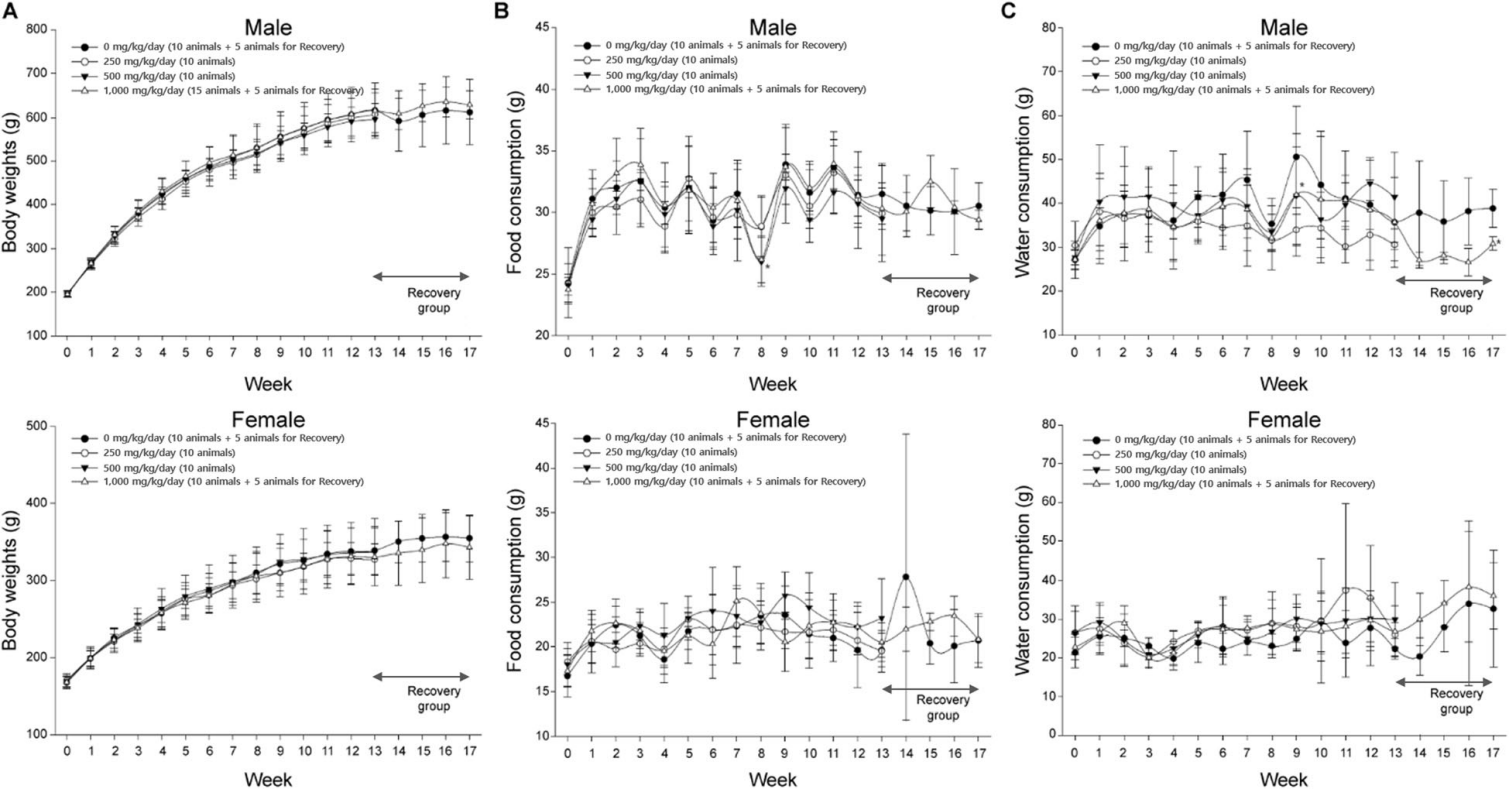
Body weights and daily food and water consumption on the repeated-dose 90-day oral toxicity study. **(a)** Body weights of male and female rats, **(b)** Mean daily food consumption of male and female rats, **(c)** Mean daily water consumption of male and female rats

We evaluated the NOAEL for repeated-dose oral toxicity of agglomerated/aggregated TiO_2_ P25 by administering the substance to SD rats for 90 days. At 24 h after the last treatment, a recovery study (5 animals in each group) with a non-dose period of 28 days was performed to confirm the persistence of toxicity without treatment. No mortality or abnormal clinical signs were detected in any of the treatment groups during the exposure and recovery periods (data not shown). Detailed clinical observations disclosed significant differences among the groups in terms of male defecation and female urination frequency (Table S4). However, these changes were temporary, and alterations in the excretion rate are common even under normal conditions with no specific fecal or urinary abnormalities. Therefore, these changes were not ascribed to the administration of the test substance. In the functional observation battery, no changes related to the agglomerated/aggregated TiO_2_ P25 were detected for sensorimotor responses, spontaneous motor activity, or grip strength in any of the treated groups (data not shown). There were no statistically significant differences among the vehicle control and dosing groups in terms of body weight during the study period (Fig. 4a). Compared to that observed in the vehicle control group, food consumption decreased in the male 500 mg kg^− 1^ d^− 1^ dosing group at week 8 (*P* < 0.05) (Fig. 4b), and water consumption declined in the male 1000 mg kg^− 1^ d^− 1^ group of the recovery study at week 17 (*P* < 0.05) (Fig. 4c). However, these discrepancies were not associated with the test substance because no dose-response correlation was found, and the alterations were either temporary or independent of subsequent related changes. No ocular abnormalities were detected in any of the animals (data not shown). The urinalysis, urine sediment test, and urine volume measurements presented with no significant differences among all treated groups (data not shown). In terms of hematology (Table 3), the PMN (neutrophil) levels were lower in the female 500 mg kg^− 1^ d^− 1^ dosing group than in the vehicle control group (*P* < 0.05). The PMNP (% neutrophils) were lower and the LYP (% lymphocyte) were higher in the female 250 mg kg^− 1^ d^− 1^, 500 mg kg^− 1^ d^− 1^, and 1000 mg kg^− 1^ d^− 1^ dosing groups than in the vehicle control group (*P* < 0.01). Nevertheless, no substantial dose-response or correlation between males and females were found. Moreover, these variations did not persist in the recovery study. Therefore, the observed changes were not associated with the administration of the agglomerated/aggregated TiO_2_ P25.

**Table 3:** Hematology of rats in repeated-dose 90-day and recovery studies, mean ± SD (number of animals)

Summary of hematological tests from the repeated-dose 90-day study																
Sex: Male																
Test item	Group(mg/kg/day)															
	G1(0)				G2(250)				G3(500)				G4(1000)			
WBC^1^(K/μL)	**6.97**	**±**	**1.2**	**(10)**	**6.46**	**±**	**1.81**	**(10)**	**8.1**	**±**	**1.39**	**(10)**	**7.22**	**±**	**1.52**	**(10)**
PMN^2^(K/μL)	**1.58**	**±**	**0.55**	**(10)**	**1.46**	**±**	**0.8**	**(10)**	**1.38**	**±**	**0.49**	**(10)**	**1.34**	**±**	**0.34**	**(10)**
EO^3^(K/μL)	**0.11**	**±**	**0.03**	**(10)**	**0.11**	**±**	**0.03**	**(10)**	**0.32**	**±**	**0.6**	**(10)**	**0.11**	**±**	**0.03**	**(10)**
BA^4^(K/μL)	**0**	**±**	**0**	**(10)**	**0**	**±**	**0**	**(10)**	**0**	**±**	**0**	**(10)**	**0**	**±**	**0**	**(10)**
LY^5^(K/μL)	**5.04**	**±**	**0.86**	**(10)**	**4.6**	**±**	**1.09**	**(10)**	**6.1**	**±**	**1.5**	**(10)**	**5.52**	**±**	**1.26**	**(10)**
MO^6^(K/μL)	**0.18**	**±**	**0.06**	**(10)**	**0.2**	**±**	**0.1**	**(10)**	**0.21**	**±**	**0.07**	**(10)**	**0.18**	**±**	**0.07**	**(10)**
LUC^7^(K/μL)	**0.07**	**±**	**0.04**	**(10)**	**0.09**	**±**	**0.05**	**(10)**	**0.09**	**±**	**0.04**	**(10)**	**0.07**	**±**	**0.03**	**(10)**
PMNP^8^(%)	**22.3**	**±**	**5.5**	**(10)**	**21.7**	**±**	**6.9**	**(10)**	**16.9**	**±**	**4.8**	**(10)**	**18.8**	**±**	**4.4**	**(10)**
EOP^9^(%)	**1.6**	**±**	**0.5**	**(10)**	**1.9**	**±**	**0.6**	**(10)**	**4.8**	**±**	**10.3**	**(10)**	**1.5**	**±**	**0.5**	**(10)**
BAP^10^(%)	**0**	**±**	**0**	**(10)**	**0**	**±**	**0**	**(10)**	**0**	**±**	**0**	**(10)**	**0**	**±**	**0**	**(10)**
LYP^11^(%)	**72.5**	**±**	**5.3**	**(10)**	**72.2**	**±**	**6.8**	**(10)**	**74.5**	**±**	**10.1**	**(10)**	**76.3**	**±**	**4.9**	**(10)**
MOP^12^(%)	**2.5**	**±**	**0.7**	**(10)**	**3**	**±**	**1**	**(10)**	**2.7**	**±**	**0.9**	**(10)**	**2.4**	**±**	**0.6**	**(10)**
LUP^13^(%)	**1**	**±**	**0.4**	**(10)**	**1.2**	**±**	**0.5**	**(10)**	**1.2**	**±**	**0.7**	**(10)**	**1**	**±**	**0.4**	**(10)**
RBC^14^(M/μL)	**8.3**	**±**	**0.36**	**(10)**	**8.37**	**±**	**0.27**	**(10)**	**8.31**	**±**	**0.37**	**(10)**	**8.41**	**±**	**0.28**	**(10)**
Hb^15^(g/dL)	**14.5**	**±**	**0.4**	**(10)**	**14.6**	**±**	**0.5**	**(10)**	**14.4**	**±**	**0.3**	**(10)**	**14.7**	**±**	**0.6**	**(10)**
RDW^16^(%)	**12.7**	**±**	**0.6**	**(10)**	**12.7**	**±**	**0.3**	**(10)**	**12.7**	**±**	**0.5**	**(10)**	**12.6**	**±**	**0.6**	**(10)**
HCT^17^(%)	**43.9**	**±**	**1.3**	**(10)**	**44.2**	**±**	**1.7**	**(10)**	**43.4**	**±**	**1.3**	**(10)**	**44.5**	**±**	**1.7**	**(10)**
MCV^18^(fL)	**52.9**	**±**	**1.6**	**(10)**	**52.8**	**±**	**1.6**	**(10)**	**52.3**	**±**	**1.6**	**(10)**	**52.9**	**±**	**1.6**	**(10)**
MCH^19^(pg)	**17.5**	**±**	**0.6**	**(10)**	**17.5**	**±**	**0.7**	**(10)**	**17.4**	**±**	**0.6**	**(10)**	**17.5**	**±**	**0.6**	**(10)**
MCHC^20^(g/dL)	**33**	**±**	**0.4**	**(10)**	**33.1**	**±**	**0.5**	**(10)**	**33.2**	**±**	**0.4**	**(10)**	**33.1**	**±**	**0.4**	**(10)**
Reti^21^(%)	**2.35**	**±**	**0.68**	**(10)**	**2.32**	**±**	**0.21**	**(10)**	**2.21**	**±**	**0.39**	**(10)**	**2.38**	**±**	**0.36**	**(10)**
MetHb^22^(%)	**2.3**	**±**	**0.1**	**(10)**	**2.4**	**±**	**0.2**	**(10)**	**2.3**	**±**	**0.4**	**(10)**	**2.3**	**±**	**0.2**	**(10)**
Heinz^23^	**0**	**±**	**0**	**(10)**	**0**	**±**	**0**	**(10)**	**0**	**±**	**0**	**(10)**	**0**	**±**	**0**	**(10)**
PLT^24^(K/μL)	**1053**	**±**	**129**	**(10)**	**1021**	**±**	**192**	**(10)**	**1108**	**±**	**119**	**(10)**	**1067**	**±**	**104**	**(10)**
MPV^25^(fL)	**6.7**	**±**	**0.6**	**(10)**	**6.7**	**±**	**0.6**	**(10)**	**6.4**	**±**	**0.8**	**(10)**	**6.7**	**±**	**0.3**	**(10)**
Sex: Female																
Test item	Group(mg/kg/day)															
	G1(0)				G2(250)				G3(500)				G4(1000)			
WBC^1^(K/μL)	**3.54**	**±**	**0.53**	**(10)**	**4.52**	**±**	**1.57**	**(10)**	**3.94**	**±**	**0.73**	**(10)**	**4**	**±**	**1.11**	**(10)**
PMN^2^(K/μL)	**0.81**	**±**	**0.26**	**(10)**	**0.65**	**±**	**0.29**	**(10)**	0.45*	**±**	**0.18**	**(10)**	**0.64**	**±**	**0.3**	**(10)**
EO^3^(K/μL)	**0.06**	**±**	**0.02**	**(10)**	**0.09**	**±**	**0.03**	**(10)**	**0.07**	**±**	**0.03**	**(10)**	**0.07**	**±**	**0.03**	**(10)**
BA^4^(K/μL)	**0**	**±**	**0**	**(10)**	**0**	**±**	**0**	**(10)**	**0**	**±**	**0**	**(10)**	**0**	**±**	**0**	**(10)**
LY^5^(K/μL)	**2.57**	**±**	**0.61**	**(10)**	**3.62**	**±**	**1.27**	**(10)**	**3.27**	**±**	**0.63**	**(10)**	**3.15**	**±**	**0.97**	**(10)**
MO^6^(K/μL)	**0.08**	**±**	**0.03**	**(10)**	**0.13**	**±**	**0.06**	**(10)**	**0.11**	**±**	**0.04**	**(10)**	**0.11**	**±**	**0.03**	**(10)**
LUC^7^(K/μL)	**0.03**	**±**	**0.02**	**(10)**	**0.04**	**±**	**0.02**	**(10)**	**0.05**	**±**	**0.01**	**(10)**	**0.04**	**±**	**0.01**	**(10)**
PMNP^8^(%)	**23.4**	**±**	**8.8**	**(10)**	14.0**	**±**	**3.7**	**(10)**	11.3**	**±**	**3.9**	**(10)**	16.1**	**±**	**5.8**	**(10)**
EOP^9^(%)	**1.7**	**±**	**0.6**	**(10)**	**2**	**±**	**0.5**	**(10)**	**1.8**	**±**	**0.9**	**(10)**	**1.9**	**±**	**0.4**	**(10)**
BAP^10^(%)	**0**	**±**	**0**	**(10)**	**0**	**±**	**0**	**(10)**	**0**	**±**	**0**	**(10)**	**0**	**±**	**0**	**(10)**
LYP^11^(%)	**71.8**	**±**	**8.7**	**(10)**	80.2**	**±**	**4.4**	**(10)**	83.1**	**±**	**4.5**	**(10)**	78.4**	**±**	**6.2**	**(10)**
MOP^12^(%)	**2.3**	**±**	**0.7**	**(10)**	**2.8**	**±**	**0.8**	**(10)**	**2.7**	**±**	**0.7**	**(10)**	**2.7**	**±**	**1.1**	**(10)**
LUP^13^(%)	**0.9**	**±**	**0.4**	**(10)**	**1**	**±**	**0.4**	**(10)**	**1.2**	**±**	**0.5**	**(10)**	**0.9**	**±**	**0.3**	**(10)**
RBC^14^(M/μL)	**7.61**	**±**	**0.28**	**(10)**	**7.56**	**±**	**0.48**	**(10)**	**7.64**	**±**	**0.34**	**(10)**	**7.6**	**±**	**0.31**	**(10)**
Hb^15^(g/dL)	**14.2**	**±**	**0.3**	**(10)**	**14.1**	**±**	**0.7**	**(10)**	**14**	**±**	**0.7**	**(10)**	**14.3**	**±**	**0.5**	**(10)**
RDW^16^(%)	**11**	**±**	**0.4**	**(10)**	**10.9**	**±**	**0.4**	**(10)**	**11**	**±**	**0.4**	**(10)**	**10.9**	**±**	**0.4**	**(10)**
HCT^17^(%)	**41.7**	**±**	**1.1**	**(10)**	**41.2**	**±**	**1.9**	**(10)**	**41.4**	**±**	**1.9**	**(10)**	**41.8**	**±**	**1.6**	**(10)**
MCV^18^(fL)	**54.9**	**±**	**1.6**	**(10)**	**54.6**	**±**	**1.7**	**(10)**	**54.2**	**±**	**1.9**	**(10)**	**55**	**±**	**1.1**	**(10)**
MCH^19^(pg)	**18.6**	**±**	**0.5**	**(10)**	**18.6**	**±**	**0.6**	**(10)**	**18.4**	**±**	**0.6**	**(10)**	**18.7**	**±**	**0.4**	**(10)**
MCHC^20^(g/dL)	**33.9**	**±**	**0.6**	**(10)**	**34.1**	**±**	**0.6**	**(10)**	**34**	**±**	**0.6**	**(10)**	**34.1**	**±**	**0.6**	**(10)**
Reti^21^(%)	**1.86**	**±**	**0.31**	**(10)**	**1.96**	**±**	**0.25**	**(10)**	**2.1**	**±**	**0.65**	**(10)**	**1.96**	**±**	**0.36**	**(10)**
MetHb^22^(%)	**2.4**	**±**	**0.3**	**(10)**	**2.4**	**±**	**0.2**	**(10)**	**2.5**	**±**	**0.2**	**(10)**	**2.3**	**±**	**0.1**	**(10)**
Heinz^23^	**0**	**±**	**0**	**(10)**	**0**	**±**	**0**	**(10)**	**0**	**±**	**0**	**(10)**	**0**	**±**	**0**	**(10)**
PLT^24^(K/μL)	**1005**	**±**	**197**	**(10)**	**1058**	**±**	**104**	**(10)**	**1052**	**±**	**106**	**(10)**	**981**	**±**	**161**	**(10)**
MPV^25^(fL)	**7**	**±**	**0.5**	**(10)**	**7.1**	**±**	**0.5**	**(10)**	**7.2**	**±**	**0.7**	**(10)**	**7**	**±**	**0.5**	**(10)**
Summary of hematological tests from the recovery study																
Test item	Sex: Male								Sex: Female							
	Group(mg/kg/day)								Group(mg/kg/day)							
	G1(0)				G4(1000)				G1(0)				G4(1000)			
WBC^1^(K/μL)	**6.27**	**±**	**1.53**	**(5)**	**5.81**	**±**	**1.39**	**(5)**	**4.04**	**±**	**0.91**	**(5)**	**3.8**	**±**	**0.86**	**(5)**
PMN^2^(K/μL)	**1.4**	**±**	**0.57**	**(5)**	**0.9**	**±**	**0.23**	**(5)**	**0.66**	**±**	**0.27**	**(5)**	**0.54**	**±**	**0.16**	**(5)**
EO^3^(K/μL)	**0.06**	**±**	**0.03**	**(5)**	**0.07**	**±**	**0.02**	**(5)**	**0.06**	**±**	**0.02**	**(5)**	**0.06**	**±**	**0.02**	**(5)**
BA^4^(K/μL)	**0**	**±**	**0**	**(5)**	**0**	**±**	**0**	**(5)**	**0**	**±**	**0**	**(5)**	**0**	**±**	**0**	**(5)**
LY^5^(K/μL)	**4.64**	**±**	**1.06**	**(5)**	**4.66**	**±**	**1.37**	**(5)**	**3.13**	**±**	**0.7**	**(5)**	**3.05**	**±**	**0.81**	**(5)**
MO^6^(K/μL)	**0.12**	**±**	**0.08**	**(5)**	**0.13**	**±**	**0.04**	**(5)**	**0.12**	**±**	**0.03**	**(5)**	**0.1**	**±**	**0.02**	**(5)**
LUC^7^(K/μL)	**0.05**	**±**	**0.02**	**(5)**	**0.05**	**±**	**0.02**	**(5)**	**0.07**	**±**	**0.04**	**(5)**	**0.04**	**±**	**0.01**	**(5)**
PMNP^8^(%)	**22**	**±**	**6.2**	**(5)**	**16.2**	**±**	**5.4**	**(5)**	**16.1**	**±**	**4.3**	**(5)**	**14.5**	**±**	**4.2**	**(5)**
EOP^9^(%)	**0.9**	**±**	**0.6**	**(5)**	**1.3**	**±**	**0.4**	**(5)**	**1.4**	**±**	**0.3**	**(5)**	**1.7**	**±**	**0.4**	**(5)**
BAP^10^(%)	**0**	**±**	**0**	**(5)**	**0**	**±**	**0.1**	**(5)**	**0**	**±**	**0**	**(5)**	**0**	**±**	**0**	**(5)**
LYP^11^(%)	**74.4**	**±**	**5.3**	**(5)**	**79.4**	**±**	**5.7**	**(5)**	**77.6**	**±**	**4.4**	**(5)**	**79.9**	**±**	**4.2**	**(5)**
MOP^12^(%)	**1.9**	**±**	**1**	**(5)**	**2.2**	**±**	**0.7**	**(5)**	**3**	**±**	**0.4**	**(5)**	**2.8**	**±**	**0.7**	**(5)**
LUP^13^(%)	**0.8**	**±**	**0.2**	**(5)**	**0.8**	**±**	**0.2**	**(5)**	**1.9**	**±**	**1.1**	**(5)**	**1**	**±**	**0.3**	**(5)**
RBC^14^(M/μL)	**8.72**	**±**	**0.41**	**(5)**	**8.38**	**±**	**0.28**	**(5)**	**7.78**	**±**	**0.63**	**(5)**	**7.75**	**±**	**0.34**	**(5)**
Hb^15^(g/dL)	**14.7**	**±**	**0.7**	**(5)**	**14.7**	**±**	**0.5**	**(5)**	**14.2**	**±**	**0.7**	**(5)**	**14.1**	**±**	**0.5**	**(5)**
RDW^16^(%)	**13.3**	**±**	**1.3**	**(5)**	**13**	**±**	**0.5**	**(5)**	**11.4**	**±**	**0.2**	**(5)**	**11.3**	**±**	**0.4**	**(5)**
HCT^17^(%)	**45.1**	**±**	**1.8**	**(5)**	**45.1**	**±**	**1.6**	**(5)**	**42.6**	**±**	**2.2**	**(5)**	**42.3**	**±**	**1.6**	**(5)**
MCV^18^(fL)	**51.8**	**±**	**1.3**	**(5)**	**53.8**	**±**	**2.1**	**(5)**	**54.8**	**±**	**2.8**	**(5)**	**54.6**	**±**	**1.9**	**(5)**
MCH^19^(pg)	**16.9**	**±**	**0.6**	**(5)**	**17.5**	**±**	**0.7**	**(5)**	**18.3**	**±**	**1**	**(5)**	**18.3**	**±**	**0.6**	**(5)**
MCHC^20^(g/dL)	**32.7**	**±**	**0.5**	**(5)**	**32.6**	**±**	**0.2**	**(5)**	**33.5**	**±**	**0.3**	**(5)**	**33.4**	**±**	**0.2**	**(5)**
Reti^21^(%)	**2.39**	**±**	**0.5**	**(5)**	**2.48**	**±**	**0.86**	**(5)**	**1.96**	**±**	**0.29**	**(5)**	**1.86**	**±**	**0.15**	**(5)**
MetHb^22^(%)	**1.7**	**±**	**0.8**	**(5)**	**2.1**	**±**	**0.9**	**(5)**	**1.2**	**±**	**0.8**	**(5)**	**1.8**	**±**	**0.8**	**(5)**
Heinz^23^	**0**	**±**	**0**	**(5)**	**0**	**±**	**0**	**(5)**	**0**	**±**	**0**	**(5)**	**0**	**±**	**0**	**(5)**
PLT^24^(K/μL)	**1091**	**±**	**52**	**(5)**	**1116**	**±**	**133**	**(5)**	**902**	**±**	**237**	**(5)**	**1068**	**±**	**186**	**(5)**
MPV^25^(fL)	**8.2**	**±**	**0.4**	**(5)**	**8.3**	**±**	**0.2**	**(5)**	**6.6**	**±**	**1.2**	**(5)**	**6.5**	**±**	**1**	**(5)**

1: White blood cell, 2: Neutrophil, 3: Eosinophil, 4: Basophil, 5: Lymphocyte, 6: Monocyte, 7: Large unstained cell, 8: Percent of neutrophil, 9: Percent of eosinophil, 10: Percent of basophil, 11: Percent of lymphocyte, 12: Percent of monocyte, 13: Percent of large unstained cell, 14: Red blood cell, 15: Hemoglobin, 16: Red cell distribution width, 17: Hematocrit, 18: Mean corpuscular volume, 19: Mean corpuscular hemoglobin, 20: Mean corpuscular hemoglobin concentration, 21: Reticulocyte, 22: Methemoglobin, 23: Heinz body, 24: Platelet, 25: Mean platelet volume

*: Significant difference compared with the vehicle control group, *P* < 0.05

**: Significant difference compared with the vehicle control group, *P* < 0.01

In the blood coagulation test (data not shown) and serum biochemistry (Table 4), there were no statistically significant differences between the vehicle control and dosing groups in the 90-day treatment and recovery studies, although relatively lower BUN (blood urea nitrogen) levels were observed in the female 1000 mg kg^− 1^ d^− 1^ group of the 90-day treatment study (*P* < 0.05). However, the difference was minor compared to that of the vehicle control group, presenting no clinical significance. In the recovery study, the Na levels in the male 1000 mg kg^− 1^ d^− 1^ dosing group were lower than those of the vehicle control group (*P* < 0.05). However, the reduced values still fit within the normal range, thus considered unrelated to the test substance.

**Table 4:** Serum biochemistry from repeated-dose 90-day and recovery studies, mean ± SD (number of animals)

Summary of serum biochemical tests from the repeated dose 90-day study																
Sex: Male																
Test item	Group(mg/kg/day)															
	G1(0)				G2(250)				G3(500)				G4(1000)			
AST^1^(IU/L)	**124**	**±**	**61**	**(10)**	**135**	**±**	**73**	**(10)**	**100**	**±**	**27**	**(10)**	**97**	**±**	**17**	**(10)**
ALT^2^(IU/L)	**38**	**±**	**14**	**(10)**	**50**	**±**	**51**	**(10)**	**30**	**±**	**3**	**(10)**	**31**	**±**	**7**	**(10)**
ALP^3^(IU/L)	**209**	**±**	**39**	**(10)**	**219**	**±**	**68**	**(10)**	**217**	**±**	**50**	**(10)**	**186**	**±**	**40**	**(10)**
BA^4^(μmol/L)	**14.1**	**±**	**6.5**	**(10)**	**20.8**	**±**	**16**	**(10)**	**13.7**	**±**	**4.2**	**(10)**	**12.8**	**±**	**4.6**	**(10)**
BIL^5^(mg/dL)	**0.04**	**±**	**0.02**	**(10)**	**0.04**	**±**	**0.02**	**(10)**	**0.04**	**±**	**0.01**	**(10)**	**0.04**	**±**	**0.02**	**(10)**
BUN^6^(mg/dL)	**14.5**	**±**	**2.7**	**(10)**	**15.4**	**±**	**2**	**(10)**	**15.7**	**±**	**1.8**	**(10)**	**13.9**	**±**	**1.5**	**(10)**
CRE^7^(mg/dL)	**0.45**	**±**	**0.05**	**(10)**	**0.45**	**±**	**0.03**	**(10)**	**0.45**	**±**	**0.05**	**(10)**	**0.46**	**±**	**0.04**	**(10)**
UA^8^(mg/dL)	**1**	**±**	**0.1**	**(10)**	**1**	**±**	**0.2**	**(10)**	**1**	**±**	**0.2**	**(10)**	**0.9**	**±**	**0.1**	**(10)**
GLU^9^(mg/dL)	**181**	**±**	**19**	**(10)**	**178**	**±**	**28**	**(10)**	**188**	**±**	**47**	**(10)**	**184**	**±**	**33**	**(10)**
CHO^10^(mg/dL)	**84**	**±**	**17**	**(10)**	**93**	**±**	**18**	**(10)**	**87**	**±**	**20**	**(10)**	**91**	**±**	**16**	**(10)**
TG^11^(mg/dL)	**73**	**±**	**39**	**(10)**	**72**	**±**	**33**	**(10)**	**75**	**±**	**30**	**(10)**	**72**	**±**	**32**	**(10)**
PRO^12^(g/dL)	**6.3**	**±**	**0.2**	**(10)**	**6.4**	**±**	**0.2**	**(10)**	**6.2**	**±**	**0.2**	**(10)**	**6.3**	**±**	**0.3**	**(10)**
ALB^13^(g/dL)	**2.4**	**±**	**0.1**	**(10)**	**2.4**	**±**	**0.1**	**(10)**	**2.4**	**±**	**0.2**	**(10)**	**2.5**	**±**	**0.1**	**(10)**
A/Gratio^14^	**0.61**	**±**	**0.04**	**(10)**	**0.6**	**±**	**0.04**	**(10)**	**0.62**	**±**	**0.08**	**(10)**	**0.64**	**±**	**0.05**	**(10)**
LDH^15^(IU/L)	**840**	**±**	**886**	**(10)**	**1037**	**±**	**736**	**(10)**	**826**	**±**	**551**	**(10)**	**742**	**±**	**448**	**(10)**
CPK^16^(U/L)	**396**	**±**	**326**	**(10)**	**466**	**±**	**287**	**(10)**	**397**	**±**	**197**	**(10)**	**369**	**±**	**182**	**(10)**
ChE^17^(U/L)	**199**	**±**	**51**	**(10)**	**181**	**±**	**27**	**(10)**	**168**	**±**	**54**	**(10)**	**203**	**±**	**87**	**(10)**
Ca^18^(mg/dL)	**9.1**	**±**	**0.3**	**(10)**	**9.4**	**±**	**0.5**	**(10)**	**9.1**	**±**	**0.4**	**(10)**	**9.1**	**±**	**0.3**	**(10)**
IP^19^(mg/dL)	**6.6**	**±**	**0.5**	**(10)**	**6.7**	**±**	**0.6**	**(10)**	**6.8**	**±**	**0.5**	**(10)**	**6.8**	**±**	**0.4**	**(10)**
Mg^20^(mg/dL)	**2**	**±**	**0.1**	**(10)**	**2.1**	**±**	**0.2**	**(10)**	**2.1**	**±**	**0.1**	**(10)**	**2**	**±**	**0.1**	**(10)**
Na^21^(mmol/L)	**137**	**±**	**5**	**(10)**	**138**	**±**	**3**	**(10)**	**138**	**±**	**6**	**(10)**	**137**	**±**	**4**	**(10)**
K^22^(mmol/L)	**4.5**	**±**	**0.3**	**(10)**	**4.7**	**±**	**0.3**	**(10)**	**4.5**	**±**	**0.3**	**(10)**	**4.6**	**±**	**0.3**	**(10)**
Cl^23^(mmol/L)	**107**	**±**	**3**	**(10)**	**107**	**±**	**2**	**(10)**	**105**	**±**	**5**	**(10)**	**110**	**±**	**5**	**(10)**
Sex: Female																
Test item	Group(mg/kg/day)															
	G1(0)				G2(250)				G3(500)				G4(1000)			
AST^1^(IU/L)	**121**	**±**	**38**	**(10)**	**91**	**±**	**16**	**(10)**	**127**	**±**	**57**	**(10)**	**108**	**±**	**27**	**(10)**
ALT^2^(IU/L)	**35**	**±**	**15**	**(10)**	**26**	**±**	**3**	**(10)**	**36**	**±**	**18**	**(10)**	**43**	**±**	**22**	**(10)**
ALP^3^(IU/L)	**118**	**±**	**27**	**(10)**	**98**	**±**	**22**	**(10)**	**88**	**±**	**15**	**(10)**	**105**	**±**	**35**	**(10)**
BA^4^(μmol/L)	**22.9**	**±**	**13.8**	**(10)**	**13.9**	**±**	**6.8**	**(10)**	**16.2**	**±**	**6.9**	**(10)**	**17.8**	**±**	**7.7**	**(10)**
BIL^5^(mg/dL)	**0.09**	**±**	**0.02**	**(10)**	**0.09**	**±**	**0.02**	**(10)**	**0.11**	**±**	**0.02**	**(10)**	**0.1**	**±**	**0.03**	**(10)**
BUN^6^(mg/dL)	**17.8**	**±**	**1.3**	**(10)**	**16.3**	**±**	**1.6**	**(10)**	**16.3**	**±**	**2.8**	**(10)**	15.0*	**±**	**2**	**(10)**
CRE^7^(mg/dL)	**0.52**	**±**	**0.04**	**(10)**	**0.56**	**±**	**0.06**	**(10)**	**0.55**	**±**	**0.05**	**(10)**	**0.54**	**±**	**0.04**	**(10)**
UA^8^(mg/dL)	**1**	**±**	**0.2**	**(10)**	**0.9**	**±**	**0.2**	**(10)**	**0.9**	**±**	**0.2**	**(10)**	**0.9**	**±**	**0.2**	**(10)**
GLU^9^(mg/dL)	**133**	**±**	**10**	**(10)**	**143**	**±**	**20**	**(10)**	**144**	**±**	**16**	**(10)**	**142**	**±**	**22**	**(10)**
CHO^10^(mg/dL)	**82**	**±**	**17**	**(10)**	**98**	**±**	**19**	**(10)**	**101**	**±**	**28**	**(10)**	**90**	**±**	**23**	**(10)**
TG^11^(mg/dL)	**42**	**±**	**13**	**(10)**	**32**	**±**	**5**	**(10)**	**44**	**±**	**25**	**(10)**	**42**	**±**	**27**	**(10)**
PRO^12^(g/dL)	**6.7**	**±**	**0.4**	**(10)**	**7**	**±**	**0.5**	**(10)**	**7**	**±**	**0.5**	**(10)**	**6.8**	**±**	**0.4**	**(10)**
ALB^13^(g/dL)	**2.8**	**±**	**0.2**	**(10)**	**3**	**±**	**0.4**	**(10)**	**3**	**±**	**0.3**	**(10)**	**2.9**	**±**	**0.3**	**(10)**
A/Gratio^14^	**0.73**	**±**	**0.03**	**(10)**	**0.74**	**±**	**0.07**	**(10)**	**0.75**	**±**	**0.08**	**(10)**	**0.75**	**±**	**0.06**	**(10)**
LDH^15^(IU/L)	**845**	**±**	**321**	**(10)**	**703**	**±**	**370**	**(10)**	**787**	**±**	**456**	**(10)**	**813**	**±**	**465**	**(10)**
CPK^16^(U/L)	**459**	**±**	**181**	**(10)**	**351**	**±**	**172**	**(10)**	**402**	**±**	**232**	**(10)**	**430**	**±**	**249**	**(10)**
ChE^17^(U/L)	**1381**	**±**	**360**	**(10)**	**1438**	**±**	**188**	**(10)**	**1315**	**±**	**226**	**(10)**	**1285**	**±**	**174**	**(10)**
Ca^18^(mg/dL)	**9.8**	**±**	**0.4**	**(10)**	**10**	**±**	**0.4**	**(10)**	**10**	**±**	**0.4**	**(10)**	**9.8**	**±**	**0.5**	**(10)**
IP^19^(mg/dL)	**6.1**	**±**	**0.5**	**(10)**	**6**	**±**	**1.2**	**(10)**	**5.7**	**±**	**0.4**	**(10)**	**5.9**	**±**	**0.5**	**(10)**
Mg^20^(mg/dL)	**2.1**	**±**	**0.1**	**(10)**	**2.2**	**±**	**0.1**	**(10)**	**2.1**	**±**	**0.1**	**(10)**	**2.2**	**±**	**0.1**	**(10)**
Na^21^(mmol/L)	**142**	**±**	**7**	**(10)**	**139**	**±**	**4**	**(10)**	**139**	**±**	**4**	**(10)**	**142**	**±**	**5**	**(10)**
K^22^(mmol/L)	**4.6**	**±**	**0.3**	**(10)**	**4.4**	**±**	**0.5**	**(10)**	**4.2**	**±**	**0.3**	**(10)**	**4.4**	**±**	**0.3**	**(10)**
Cl^23^(mmol/L)	**100**	**±**	**5**	**(10)**	**102**	**±**	**2**	**(10)**	**102**	**±**	**2**	**(10)**	**103**	**±**	**5**	**(10)**
Summary of serum biochemical tests from the recovery study																
	Sex: Male								Sex: Female							
Test item	Group(mg/kg/day)								Group(mg/kg/day)							
	G1(0)				G4(1000)				G1(0)				G4(1000)			
AST^1^(IU/L)	**126**	**±**	**42**	**(5)**	**133**	**±**	**21**	**(5)**	**135**	**±**	**36**	**(5)**	**143**	**±**	**56**	**(5)**
ALT^2^(IU/L)	**51**	**±**	**31**	**(5)**	**42**	**±**	**10**	**(5)**	**30**	**±**	**8**	**(5)**	**35**	**±**	**15**	**(5)**
ALP^3^(IU/L)	**184**	**±**	**43**	**(5)**	**183**	**±**	**20**	**(5)**	**80**	**±**	**17**	**(5)**	**75**	**±**	**25**	**(5)**
BA^4^(μmol/L)	**18.4**	**±**	**11.4**	**(5)**	**23.3**	**±**	**7.8**	**(5)**	**22.7**	**±**	**13.8**	**(5)**	**23.4**	**±**	**22**	**(5)**
BIL^5^(mg/dL)	**0.08**	**±**	**0.04**	**(5)**	**0.06**	**±**	**0.02**	**(5)**	**0.08**	**±**	**0.01**	**(5)**	**0.11**	**±**	**0.02**	**(5)**
BUN^6^(mg/dL)	**15.2**	**±**	**1.9**	**(5)**	**15**	**±**	**1**	**(5)**	**16.9**	**±**	**2.6**	**(5)**	**18.3**	**±**	**2.8**	**(5)**
CRE^7^(mg/dL)	**0.49**	**±**	**0.04**	**(5)**	**0.49**	**±**	**0.03**	**(5)**	**0.5**	**±**	**0.07**	**(5)**	**0.51**	**±**	**0.03**	**(5)**
UA^8^(mg/dL)	**1**	**±**	**0.2**	**(5)**	**0.9**	**±**	**0.2**	**(5)**	**0.7**	**±**	**0.1**	**(5)**	**0.7**	**±**	**0.2**	**(5)**
GLU^9^(mg/dL)	**186**	**±**	**35**	**(5)**	**179**	**±**	**35**	**(5)**	**128**	**±**	**15**	**(5)**	**140**	**±**	**10**	**(5)**
CHO^10^(mg/dL)	**60**	**±**	**17**	**(5)**	**77**	**±**	**25**	**(5)**	**98**	**±**	**15**	**(5)**	**105**	**±**	**13**	**(5)**
TG^11^(mg/dL)	**66**	**±**	**29**	**(5)**	**61**	**±**	**27**	**(5)**	**47**	**±**	**14**	**(5)**	**51**	**±**	**16**	**(5)**
PRO^12^(g/dL)	**6.3**	**±**	**0.3**	**(5)**	**6.2**	**±**	**0.3**	**(5)**	**6.8**	**±**	**0.5**	**(5)**	**7**	**±**	**0.4**	**(5)**
ALB^13^(g/dL)	**2.5**	**±**	**0.1**	**(5)**	**2.4**	**±**	**0.1**	**(5)**	**3**	**±**	**0.3**	**(5)**	**3.1**	**±**	**0.2**	**(5)**
A/Gratio^14^	**0.66**	**±**	**0.05**	**(5)**	**0.62**	**±**	**0.02**	**(5)**	**0.81**	**±**	**0.05**	**(5)**	**0.81**	**±**	**0.04**	**(5)**
LDH^15^(IU/L)	**897**	**±**	**524**	**(5)**	**803**	**±**	**391**	**(5)**	**1148**	**±**	**575**	**(5)**	**851**	**±**	**385**	**(5)**
CPK^16^(U/L)	**344**	**±**	**164**	**(5)**	**360**	**±**	**180**	**(5)**	**561**	**±**	**261**	**(5)**	**387**	**±**	**169**	**(5)**
ChE^17^(U/L)	**188**	**±**	**42**	**(5)**	**204**	**±**	**38**	**(5)**	**1678**	**±**	**482**	**(5)**	**1363**	**±**	**373**	**(5)**
Ca^18^(mg/dL)	**9.5**	**±**	**0.6**	**(5)**	**9.3**	**±**	**0.2**	**(5)**	**9.8**	**±**	**0.4**	**(5)**	**9.7**	**±**	**0.3**	**(5)**
IP^19^(mg/dL)	**6.2**	**±**	**0.9**	**(5)**	**6.5**	**±**	**1**	**(5)**	**6.8**	**±**	**1**	**(5)**	**7.2**	**±**	**1.2**	**(5)**
Mg^20^(mg/dL)	**2.2**	**±**	**0.2**	**(5)**	**2.1**	**±**	**0**	**(5)**	**2.2**	**±**	**0.2**	**(5)**	**2.4**	**±**	**0.3**	**(5)**
Na^21^(mmol/L)	**144**	**±**	**3**	**(5)**	140*	**±**	**2**	**(5)**	**139**	**±**	**1**	**(5)**	**139**	**±**	**1**	**(5)**
K^22^(mmol/L)	**4.8**	**±**	**0.4**	**(5)**	**4.8**	**±**	**0.2**	**(5)**	**4.3**	**±**	**0.2**	**(5)**	**4.3**	**±**	**0.2**	**(5)**
Cl^23^(mmol/L)	**106**	**±**	**2**	**(5)**	**104**	**±**	**1**	**(5)**	**103**	**±**	**2**	**(5)**	**101**	**±**	**3**	**(5)**

1: Aspartate aminotransferase, 2: Alanine aminotransferase, 3: Alkaline phosphatase, 4: Bile acid, 5: Total bilirubin, 6: Blood urea nitrogen, 7: Creatinine, 8: Uric acid, 9: Glucose, 10: Total cholesterol, 11: Triglyceride, 12: Total protein, 13: Albumin, 14: Albumin/Globulin ratio, 15: Lactate dehydrogenase, 16: Creatine phosphokinase, 17: Cholinesterase, 18: Calcium, 19: Inorganic phosphorus, 20: Magnesium, 21: Sodium, 22: Potassium, 23: Chloride

*: Significant difference compared with the vehicle control group value, *P* < 0.05

There were no abnormal gross findings in any of the animals in the 90-day treatment and recovery studies at necropsy (Table S5). For the 90-day treatment study, there were no significant differences in absolute organ weight between the vehicle control and treated groups (Table S6). Higher absolute pituitary weights were measured in the male 1000 mg kg^− 1^ d^− 1^ group of the recovery study (*P* < 0.05), lower absolute uterine weights were determined in the female 1000 mg kg^− 1^ d^− 1^ group of the recovery study (*P* < 0.05), and higher relative liver weights were found in the female 1000 mg kg^− 1^ d^− 1^ group of the recovery study than in the vehicle control group (*P* < 0.05) (Table S7). As these alterations were intermittent and restricted to the recovery study, they were not related to the agglomerated/aggregated TiO_2_ P25. The gross and histopathological findings revealed no abnormalities at necropsy. However, the histopathological examination (Table 5) disclosed lesions in the vehicle control and the 1000 mg kg^− 1^ d^− 1^ groups of both sexes. These included cysts or hyaline droplets on the inner stripe in the kidney, focal tubular degeneration or regeneration/degeneration and mononuclear cell infiltration to the interstitium of the kidney cortex, unilateral pyelitis, ectopic thymus or ultimobranchial cysts in the thymus, cardiomyopathy, and focal/multifocal inflammatory cell infiltration to the bronchiolo-alveoli. Nevertheless, the lesions observed in the lungs were sporadic and their frequency of occurrence did not differ significantly from that of the vehicle control group. Similarly, the occurrences of other lesions were isolated and spontaneous. For this reason, they were not considered to be associated with the agglomerated/aggregated TiO_2_ P25.

**Table 5:** Histopathological findings of male and female rats from the repeated-dose 90-day study and recovery study

Summary of histopathological findings from the repeated-dose 90-day study							
Sex: Male							
Organs	Signs		Group(mg/kg/day)				
			G1(0)			G4(1000)	
			N	%		N	%
Liver	No remarkable lesions		10/10	100		10/10	100
Kidney	No remarkable lesions		9/10	90		9/10	90
	Remarkable lesions		1/10	10		1/10	10
	- Cyst, inner stripe	+	1/10	10		0/10	0
	- Degeneration, tubular, focal, cortex	±	1/10	10		0/10	0
	- Pyelitis, unilateral	++	0/10	0		1/10	10
Adrenal gl.	No remarkable lesions		10/10	100		10/10	100
Urinary bladder	No remarkable lesions		10/10	100		10/10	100
Spleen	No remarkable lesions		10/10	100		10/10	100
Pancreas	No remarkable lesions		10/10	100		10/10	100
Thymus	No remarkable lesions		10/10	100		10/10	100
Thyroid	No remarkable lesions		9/10	90		9/10	90
	Remarkable lesions		1/10	10		1/10	10
	- Ectopic thymus	√	1/10	10		1/10	10
Parathyroid	No remarkable lesions		10/10	100		10/10	100
Trachea	No remarkable lesions		10/10	100		10/10	100
Esophagus	No remarkable lesions		10/10	100		10/10	100
Tongue	No remarkable lesions		10/10	100		10/10	100
Lung	No remarkable lesions		6/10	60		7/10	70
	Remarkable lesions		4/10	40		3/10	30
	- Cell infiltration, inflammatory, bronchioloalveolar	±	4/10	40		3/10	30
Heart	No remarkable lesions		10/10	100		9/10	90
	Remarkable lesions		0/10	0		1/10	10
	- Cardiomyopathy	±	0/10	0		1/10	10
Aorta	No remarkable lesions		10/10	100		10/10	100
Submandibular LN	No remarkable lesions		10/10	100		10/10	100
Mesenteric LN	No remarkable lesions		10/10	100		10/10	100
Sex: Female							
Organs	Signs		Group(mg/kg/day)				
			G1(0)			G4(1000)	
			N	%		N	%
Liver	No remarkable lesions		10/10	100		9/10	90
	Remarkable lesions		0/10	0		1/10	10
	- Necrosis, focal	+	0/10	0		1/10	10
Kidney	No remarkable lesions		9/10	90		9/10	90
	Remarkable lesions		1/10	10		1/10	10
	- Cyst, inner stripe	±	1/10	10		0/10	0
	- Cell infiltration, mononuclear, focal, cortex, interstitial	±	1/10	10		0/10	0
	- Hyaline droplets, inner stripe	±	0/10	0		1/10	10
Adrenal gl.	No remarkable lesions		10/10	100		10/10	100
Urinary bladder	No remarkable lesions		10/10	100		10/10	100
Spleen	No remarkable lesions		10/10	100		10/10	100
Pancreas	No remarkable lesions		10/10	100		10/10	100
Thymus	No remarkable lesions		10/10	100		10/10	100
Thyroid	No remarkable lesions		9/10	90		9/10	90
	Remarkable lesions		1/10	10		1/10	10
	- Ultimobranchial cyst	√	1/10	10		1/10	10
Parathyroid	No remarkable lesions		9/10	90		10/10	100
Trachea	No remarkable lesions		10/10	100		10/10	100
Esophagus	No remarkable lesions		10/10	100		10/10	100
Tongue	No remarkable lesions		10/10	100		10/10	100
Lung	No remarkable lesions		8/10	80		7/10	70
	Remarkable lesions		2/10	20		3/10	30
	- Cell infiltration, inflammatory, bronchioloalveolar	±	2/10	20		2/10	20
		+	0/10	0		1/10	10
Heart	No remarkable lesions		10/10	100		9/10	90
	Remarkable lesions		0/10	0		1/10	10
	- Cardiomyopathy	±	0/10	0		1/10	10
Aorta	No remarkable lesions		10/10	100		10/10	100
Submandibular LN	No remarkable lesions		10/10	100		10/10	100
Mesenteric LN	No remarkable lesions		10/10	100		10/10	100
Summary of histopathological findings from the recovery study							
Sex: Male							
Organs	Signs			Group(mg/kg/day)			
				G1(0)		G4(1000)	
				N	%	N	%
Liver	No remarkable lesions			5/5	100	5/5	100
Kidney	No remarkable lesions			4/5	80	5/5	100
	Remarkable lesions			1/5	20	0/5	0
	- Regeneration/degeneration, tubular, cortex	±		1/5	20	0/5	0
Adrenal gl.	No remarkable lesions			5/5	100	5/5	100
Urinary bladder	No remarkable lesions			5/5	100	5/5	100
Spleen	No remarkable lesions			5/5	100	5/5	100
Pancreas	No remarkable lesions			5/5	100	5/5	100
Thymus	No remarkable lesions			5/5	100	5/5	100
Thyroid	No remarkable lesions			5/5	100	5/5	100
Parathyroid	No remarkable lesions			5/5	100	5/5	100
Trachea	No remarkable lesions			5/5	100	5/5	100
Esophagus	No remarkable lesions			5/5	100	5/5	100
Tongue	No remarkable lesions			5/5	100	5/5	100
Lung	No remarkable lesions			1/5	20	2/5	40
	Remarkable lesions			4/5	80	3/5	60
	- Cell infiltration, inflammatory, bronchioloalveolar	±		3/5	60	3/5	60
		+		1/5	20	0/5	0
Heart	No remarkable lesions			4/5	80	4/5	80
	Remarkable lesions			1/5	20	1/5	20
	- Cardiomyopathy	±		1/5	20	1/5	20
Aorta	No remarkable lesions			5/5	100	5/5	100
Submandibular LN	No remarkable lesions			5/5	100	5/5	100
Mesenteric LN	No remarkable lesions			5/5	100	5/5	100
Salivary gl. submandibular	No remarkable lesions			5/5	100	5/5	100
Salivary gl. sublingual	No remarkable lesions			5/5	100	5/5	100
Salivary gl. parotid	No remarkable lesions			5/5	100	5/5	100
Sex: Female							
Organs	Signs			Group(mg/kg/day)			
				G1(0)		G4(1000)	
				N	%	N	%
Liver	No remarkable lesions			5/5	100	5/5	100
Kidney	No remarkable lesions			5/5	100	5/5	100
Adrenal gl.	No remarkable lesions			5/5	100	5/5	100
Urinary bladder	No remarkable lesions			5/5	100	5/5	100
Spleen	No remarkable lesions			5/5	100	5/5	100
Pancreas	No remarkable lesions			5/5	100	5/5	100
Thymus	No remarkable lesions			5/5	100	5/5	100
Thyroid	No remarkable lesions			5/5	100	5/5	100
Parathyroid	No remarkable lesions			5/5	100	5/5	100
Trachea	No remarkable lesions			5/5	100	5/5	100
Esophagus	No remarkable lesions			5/5	100	5/5	100
Tongue	No remarkable lesions			5/5	100	5/5	100
Lung	No remarkable lesions			3/5	60	3/5	60
	Remarkable lesions			2/5	40	2/5	40
	- Cell infiltration, inflammatory, bronchioloalveolar	±		1/5	20	2/5	40
		+		1/5	20	0/5	0
Heart	No remarkable lesions			5/5	100	5/5	100
Aorta	No remarkable lesions			5/5	100	5/5	100
Submandibular LN	No remarkable lesions			5/5	100	5/5	100
Mesenteric LN	No remarkable lesions			5/5	100	5/5	100
Salivary gl. submandibular	No remarkable lesions			5/5	100	5/5	100
Salivary gl. sublingual	No remarkable lesions			5/5	100	5/5	100
Salivary gl. parotid	No remarkable lesions			5/5	100	5/5	100

*N* Number of animals with the signs/Number of examined animals

±: minimal, +: mild, ++: moderate, √: present, gl. = gland, LN = lymph node

Therefore, this study clearly showed that even moderately prolonged exposure to agglomerated/aggregated TiO_2_ P25 (approximately 180 nm) via oral ingestion is highly unlikely to induce adverse effects or toxic reactions in rodents. Table 6 summarizes the data on the significant differences.

**Table 6:** Summarized data on the significant differences in analysis results

Period	Significant differences (vs. G1)	Results
**28-day**	• **Urinalysis**	• No dose-response correlation • Within biological normal ranges • Temporary or isolated symptoms without subsequent changes → No relationship with the test substance
	- SG (male – G2, 3, 4)	
	• **Hematological values**	
	- LY (female – G2, 3, 4) ↓	
	- MCV (female – G2) ↑	
	• **Serum biochemical values**	
	- Cl (female – G3) ↑	
	• **Absolute organ weight**	
	- Right adrenal glands (male – G2, 4) ↓	
	- Livers (female – G2) ↑	
**90-day**	• **Detailed clinical observations**	
	- Defecation (male in week-4)	
	- Urination (female in week-12)	
	• **Food consumption** (male in week-8 – G3) ↓	
	• **Hematological values**	
	- NE (female – G3) ↓	
	- NEP (female – G2, 3, 4) ↓	
	- LYP (female – G2, 3, 4) ↑	
	• **Serum biochemical values**	
	- BUN (female – G4) ↓	
**Recovery (for 28 days)**	• **Water intake** (male in week-17 – G4) ↓	
	• **Serum biochemical values**	
	- Na (male – G4) ↓	
	• **Absolute organ weight**	
	- Pituitary glands (male – G4) ↑	
	- Uterus (female – G4) ↓	
	• **Relative organ weight**	
	- Liver (female – G4) ↑	

## Discussion

Concerns about the potential risks of nanoscience and nanotechnology to human health are growing as the use of nano-sized materials in consumer products increase. TiO_2_ is one of the most used nanomaterials currently, and several reports have been published on the acute and subchronic oral toxicity of TiO_2_. However, based on previous studies and on our results, it is unclear whether TiO_2_ is toxic.

In the present study, we investigated the subacute and subchronic toxicity of the agglomerated/aggregated TiO_2_ P25 administered orally to rats (250 mg kg^− 1^, 500 mg kg^− 1^, or 1000 mg kg^− 1^) at 24 h intervals for 28 and 90 days; a recovery study with a non-dosing period of 28 days was also conducted to confirm the persistence of toxicity without treatment at study termination, in accordance with the Organization for Economic Cooperation and Development (OECD) 407 and 408 procedures [16, 17]. In the case of the subchronic studies with longer exposure periods than subacute studies, a recovery phase (non-dosing period) is necessary to ascertain that the toxicities observed at the end of the dosing phase are partially or completely reversible. Additionally, as the physicochemistry of the nanoparticles must be evaluated as part of the toxicity test, we characterized the agglomerated/aggregated TiO_2_ P25 by DLS and TEM. Based on a previous study, we selected 5 mM sodium phosphate buffer (pH 8.0) as the vehicle to obtain the most stable dispersion stability [18]. The dispersion protocol developed by the National Institute of Standards and Technology [19, 20] was used, and the dispersed particles were approximately 180 nm in size. The hydrodynamic diameters indicated that the primary TiO_2_ nanoparticles aggregated and agglomerated upon dispersal in the vehicle. No systemic toxicological effects were related with the agglomerated/aggregated TiO_2_ P25 in the repeated-dose 28-day and 90-day oral toxicity and 28-day recovery studies in SD rats under the experimental conditions used. Therefore, the NOAEL of the agglomerated/aggregated TiO_2_ P25 was identified as 1000 mg kg^− 1^ d^− 1^, and this test substance was not detected in the target organs.

These results are consistent with the results of previous publications. In the acute oral toxicity study of TiO_2_, Warheit et al. showed only low potential health risks in mammals or aquatic species acutely exposed to ultrafine TiO_2_ particles [21], and a fixed 5 g kg^− 1^ body weight dose of TiO_2_ suspension showed no obvious acute toxicity after two weeks [9]. In the subchronic oral toxicity study, Warheit et al. reported no significant oral toxicity induced by the nanoscale component of the TiO_2_ test material in the 90-day study [22]; furthermore, TiO_2_ particles consisting of 80% anatase and 20% rutile displayed an extremely low absorption rate in the liver, spleen, kidney, and brain tissues [23].

As mentioned above, TiO_2_-induced toxicity was not detected after acute and subchronic oral administration, but other studies have reported the opposite. Tassinari et al. explored possible effects of short-term (5 days) oral exposure to anatase TiO_2_ nanoparticles (0, 1, 2 mg/kg b.w) on the reproductive and endocrine systems of rats, and reported that the Ti levels were increased in the spleen and ovaries [24]. Moreover, Dasal et al. stated that oral administration of TiO_2_ nanoparticles (< 100 nm diameter) may induce hepatic and renal toxicity in experimental rats at 14 days post-exposure [25]. Moreover, in a 90-day study on the exposure of rats to TiO_2_ nanoparticles (intragastric administration: 2.5, 5, 10 mg/kg b.w.), the authors showed spleen injury and alteration of cytokine expression [26]. Another long-term (90-day) oral toxicity study (2.5, 5, 10 mg/kg b.w) in mice showed ovarian damage, oxidative stress, testicular lesions, and sperm malformations [27, 28]. Recent reports have confirmed the presence of agglomerated/aggregated TiO_2_ particles (85 to 720 nm) in post-mortem human liver and spleen, where 24% of the particles were 100 nm or less in size. For these reasons, health risks associated with liver damage due to TiO_2_ particles cannot be excluded [29].

Similarly, millions of tons of E171, a white pigment, are used in food each year, which can have a much greater effect than P25 on the environment and on human exposure [30]. The primary particle size of E171 is 60–300 nm, of which 10–15% is < 100 nm [31]. Recently, much attention has been paid to the safety of E171, with numerous articles being published on this issue. Some research groups have reported that exposure to E171 containing a mixture of micro- and nano-sized particles can induce ROS generation and DNA damage in in vitro models [32]; others have reported on changes in gene expression and impaired immune homeostasis occurred in the colons of BALB/c mice and promotion of aberrant crypt development in the rat colon after short-term oral administration [33, 34].

As the aforementioned conflicting results show, there is an ongoing debate about the safety of TiO_2_. Recently, France has decided to ban the use of E171, which is mainly used as a food additive, from 2020. Much of the concern is related to the unclear identification of their hazards as additives. Clearly, reliable results from well-designed toxicology tests are necessary. We aim to perform more studies in the future to investigate subchronic oral toxicity of E171.

## Conclusions

In our study, the subacute and subchronic oral toxicity of agglomerated/aggregated TiO_2_ P25 (approximately 180 nm) were investigated in rats according to the standard procedure (OECD Guidelines, No. 407 and 408) for testing chemicals. The NOAEL of the agglomerated/aggregated TiO_2_ P25 was 1000 mg kg^− 1^ d^− 1^. Although there were significant differences in some results, they were unrelated to the test substance. Collectively, the results from our studies can contribute to future safety assessment of TiO_2_ materials in humans.

## Methods

### TiO_2_ particles

TiO_2_ nanoparticles (AEROXIDE^Ⓡ^ TiO_2_ P25, KRISS CRM 301–03-001, anatase/rutile (80/20), 99.9%; average primary particle size range 14–21 nm) were purchased from Evonik Industries AG (Essen, Germany). TiO_2_ nanoparticles were in the form of a white hydrophilic powder without surface modification and were stored at room temperature. TiO_2_ nanoparticle suspensions were prepared by dispersing the particles in 5 mM sodium phosphate buffer (pH 8.0; Sigma-Aldrich Corp., St. Louis, MO, USA) followed by sonication (Branson Ultrasonics 450D; Thermo Fisher Scientific, Waltham, MA, USA) at 50 W for 17.5 min in an ice water bath to prevent the suspensions from overheating.

### Particle characterization

The hydrodynamic diameters of the particles in 5 mM sodium phosphate buffer were measured by DLS using a Zetasizer Nano ZS90 (Malvern Panalytical Ltd., Malvern, UK) and were confirmed by TEM using the FEI Tecnai F30, operated at an acceleration voltage of 300 kV. The pH value of the prepared vehicle was measured with a pH meter (Orion Star A210; Thermo Fisher Scientific, Waltham, MA, USA) and the pH range was confirmed to be 8.05–8.11.

### Animals

Pathogen-free SD rats [Crl:CD(Sprague-Dawley)] were purchased from Orient Bio Inc. (Seongnam, Korea). Healthy young adult animals (males and non-pregnant females) were acclimated and closely monitored for 6 days after arrival in the SPF animal facility area, and were randomly assigned to the control and treatment groups. Only animals with the best appearance were selected for subsequent testing. The body weights of the male and female rats were 210–232 g and 157–185 g, respectively, at the time of the first administration in the repeated-dose 28-day experiment. The body weights of the male and female rats were 185–207 g and 149–183 g, respectively, at the time of the first administration in the repeated-dose 90-day experiment. Rats were housed two per cage in an environmentally controlled room at 22.9 ± 0.5 °C and relative humidity of 54.3 ± 4.2%. The room air was replaced 10–15× per hour. Lighting was set to a 12-h light/dark cycle (on at 08 h00 and off at 20 h00).

### Experimental design

One hundred and forty healthy adult SD rats were used in this study (Table S8). For the repeated-dose 28-day experiment, the animals were randomly divided into four groups, each consisting of five animals per sex. For the repeated-dose 90-day experiment, the animals were randomly assigned to four groups. Each group consisted of 10–15 animals per sex. One group was administered 5 mM sodium phosphate buffer by gavage and served as the vehicle control group (G1). The three remaining groups received one of three agglomerated/aggregated TiO_2_ P25 dosages by gavage (250 mg kg^− 1^ d^− 1^ (G2), 500 mg kg^− 1^ d^− 1^ (G3), and 1000 mg kg^− 1^ d^− 1^ (G4)). The dosing volume was 10 mL kg^− 1^ body weight. The agglomerated/aggregated TiO_2_ P25 were administered every morning for either 28 days or 90 days.

This study was performed in compliance with Good Laboratory Practices (GLP) and the OECD Guidelines No. 407 and 408 and was approved by the Institutional Animal Care and Use Committee (IACUC) of Korea Conformity Laboratories.

### Clinical observations, body weight, and food consumption

Detailed clinical observations were made once on all surviving animals before the onset of administration. Functional observations were conducted during the last week of treatment for the 90-day treated study and during the last week of observation for the recovery study. Functional observations were performed within 6 h after administration. Individual animal weights were recorded at acquisition and grouping, before administration, once weekly during the study, and before necropsy. Food consumption was measured immediately before the first administration and once weekly during the study. To calculate daily food intake, the food ration in each cage was weighed the day before the body weight measurement, and orts (leftover food) were measured on the day of the body weight measurement. Food consumption per animal was recalculated according to the average individual consumption (g rat^− 1^ d^− 1^), and water intake was measured immediately before the first administration and once weekly during the study. The measurement and calculation of the water intake per animal were consistent with those for the food consumption.

### Hematology and clinical biochemistry

All animals were fasted overnight before necropsy, but water was provided ad libitum. At necropsy, the rats were anesthetized with isoflurane. Blood samples were extracted from the abdominal aorta using a syringe and collected in EDTA-K2 tubes (Microtainer®; Becton, Dickinson, and Company, Franklin Lakes, NJ, USA), 3.2% sodium citrate tubes (Vacuette®; Greiner Bio-One, Kremsmünster, Austria), and serum-separating tubes (Insepack®; Sekisui Diagnostics, Lexington, MA, USA). In certain cases, blood samples were collected from the jugular vein to measure the methemoglobin concentration within 6 h after administration on the last day of treatment. For the recovery study, the blood samples were drawn from the abdominal aorta at necropsy and stored in heparin tubes (20–30 IU mL^-l^). Blood collected in the EDTA-K2 tube was analyzed with a hematology analyzer (Advia 2120; Siemens Limited, Dublin, Ireland). Methemoglobin concentrations were determined with a blood gas analyzer (GEM Premier 4000; Instrumentation Laboratory Company, Bedford, MA, USA). Erythrocytes with Heinz bodies were counted after supravital staining. Blood biochemistry was analyzed with a biochemistry analyzer (Hitachi 7180; Hitachi, Chiyoda, Tokyo, Japan). Serum was isolated and collected by centrifugation in a serum-separating tube at 3000 rpm for 10 min.

### Necropsy and histopathology

After administration, necropsies were conducted on all surviving animals, and complete post-mortem examinations were performed on all organs. All organs were harvested, and some organs were weighed immediately after extraction (Table S9). Excised organs were fixed in 10% neutral phosphate-buffered formalin. Testes and epididymis were fixed in Bouin’s solution, and eyes were fixed in Davidson’s solution. Bilateral organs were fixed and organs with macroscopically abnormal lesions were preserved. Thin sections were made from all the preserved organs and tissues of the vehicle control and high-dose groups, mounted on histology slides, and examined histopathologically by hematoxylin and eosin (H&E) staining.

### Statistical analysis

Statistical differences among the vehicle control and dosing groups were analyzed by parametric or nonparametric multiple comparison methods. Differences were considered statistically significant at *P* < 0.05. The incidence rate was represented as a percentage. Statistical analysis was performed with SPSS for Windows v. 12.0 (IBM Corp., Armonk, NY, USA) and in compliance with the standard operating procedures of the testing facility.

## Supplementary information

**Additional file 1: Table S1.** Urinalysis of male and female rats in the 28-day treatment study. **Table S2.** Gross findings of male and female rats in the 28-day treatment study. **Table S3.** Absolute organ weights of rats in 28-day treatment study, mean ± SD (number of animals). **Table S4.** Detailed clinical observations of male and female rats in the 90-day treatment study. **Table S5.** Gross findings of rats in the (A) 90-day treatment and (B) recovery studies. **Table S6.** Absolute organ weights of rats in 90-day treatment study, mean ± SD (number of animals). **Table S7.** Absolute organ weight of rats in the recovery study, mean ± SD (number of animals). **Table S8.** Group description for the repeated-dose 28-day and 90-day oral toxicity studies. **Table S9.** List of collected organs

## Data Availability

The datasets used and/or analyzed during the current study are available from the corresponding author on reasonable request.

## Abbreviations

BUN: Blood urea nitrogen
DLS: Dynamic light scattering
TEM: Transmission electron microscopy
LY: Lymphocyte
LYP: Lymphocyte percentage
MCV: Mean corpuscular volume
PMN: Neutrophil
PMNP: Neutrophil percentage
NOAEL: No observed adverse effect level
TiO_2_: Nanoparticles, titanium dioxide nanoparticles
